# Supplementary material for: Length Polymorphism and Methylation Status of UPS29 Minisatellite of the ACAP3 Gene as Molecular Biomarker of Epilepsy. Sex Differences in Seizure Types and Symptoms
Source: Int J Mol Sci. 2020 Dec 2;21(23):9206. doi: 10.3390/ijms21239206 (PMC7730309; doi:10.3390/ijms21239206)
Supplement: Supplementary file 1 [file ijms-21-09206-s001.zip › UPS29_Supplementary Materials.docx]

*Suchkova I.O, Borisova E.V., Patkin E.L.*

*Association Analysis of Length Polymorphism of UPS29 Minisatellite of the ACAP3 Gene and its CCGG-Methylation Status in Patients with Symptomatic and Cryptogenic Epilepsy.*

**Supplementary Materials**

[Table S1: DNA stats results of UPS29 (allele of 17 repeats, 745 nt) 2](#_Toc42879198)

[Table S2: Results of search for nucleotide sequences that homologous to human UPS29 minisatellite in hominids 4](#_Toc42879199)

[Table S3: Sequencing results of pcr products of human UPS29 minisatellite. 10](#_Toc42879200)

[Table S4: ACAP3 protein structure 18](#_Toc42879201)

[Table S5: Some human genes from 1p36-p32, the products of which involve in the formation, functioning and pathological changes of the nervous and cardiovascular systems 18](#_Toc42879202)

[Table S6: High- and lower-confidence human imprinted gene candidates from 1p36.33-p36.32 22](#_Toc42879203)

[Table S7: PCR products size of the UPS29 alleles detected in the residents of st. petersburg (russian federation), ethnic origin - caucasian 24](#_Toc42879204)

[Figure S1: *In silico* analysis of UPS29 (search of potential sites of DNA instability and conserved domains). 10](#_Toc42877492)

[Figure S2: Schematic of UPS29 alleles according to sequencing results. 12](#_Toc42877493)

[Figure S3: ACAP3 expression in cells. 13](#_Toc42877494)

[Figure S4: ACAP3 mRNA and protein expression in tissue. 14](#_Toc42877495)

[Figure S5: ACAP3 protein expression in tissue. 15](#_Toc42877496)

[Figure S6: ACAP3 mRNA expression in tissue. 16](#_Toc42877497)

[Figure S7: ACAP3 mRNA expression in brain. 16](#_Toc42877498)

[Figure S8: ACAP3 mRNA expression in blood cells. 17](#_Toc42877499)

[Figure S9: Scheme of the location of potential sites of the interaction of transcription factors with UPS29. 22](#_Toc42877500)

[Figure S10: Hypothetical scheme explaining the cases of absence and presence of epilepsy (or Parkinson's disease) in individuals with short or long UPS29 alleles, taking into account the predicted imprinted *ACAP3* gene. 23](#_Toc42877501)

*******

**Table S1**: DNA stats results of UPS29 (allele of 17 repeats, 745 nt).

**Table S2**: Results of search for nucleotide sequences that homologous to human UPS29 minisatellite in hominids.

**Table S3**: Sequencing results of PCR products of human UPS29 minisatellite.

**Table S4**: ACAP3 protein structure.

**Table S5**: Some human genes from 1p36-p32, the products of which involve in the formation, functioning and pathological changes of the nervous and cardiovascular systems.

**Table S6**: High- and lower-confidence human imprinted gene candidates from 1p36.33-p36.32

**Table S7**: PCR products size of the UPS29 alleles detected in the residents of St. Petersburg (Russian Federation), ethnic origin – Caucasian

**Figure S1**: *In silico* analysis of UPS29 (search of potential sites of DNA instability and conserved domains).

**Figure S2**: Schematic of UPS29 alleles according to sequencing results.

**Figure S3**: ACAP3 expression in cells.

**Figure S4**: ACAP3 mRNA and protein expression in tissue.

**Figure S5**: ACAP3 protein expression in tissue.

**Figure S6**: ACAP3 mRNA expression in tissue.

**Figure S7**: ACAP3 mRNA expression in brain.

**Figure S8**: ACAP3 mRNA expression in blood cells.

**Figure S9**: Scheme of the location of potential sites of the interaction of transcription factors with UPS29.

**Figure S10**: Hypothetical scheme explaining the cases of absence and presence of epilepsy (or Parkinson's disease) in individuals with short or long UPS29 alleles, taking into account the predicted imprinted *ACAP3* gene.

Table S1. DNA stats results of UPS29 (allele of 17 repeats, 745 nt) (Sequence Manipulation Suite, <https://www.bioinformatics.org/sms2/genbank_fasta.html>, accessed 21.05.2020).

| **Pattern**  **(nucleotide)** | **Times found** | **Percentage** |
| --- | --- | --- |
| a | 118 | 15.84 |
| g | 208 | 27.92 |
| c | 332 | 44.56 |
| t | 87 | 11.68 |
| gg | 65 | 8.74 |
| ga | 16 | 2.15 |
| gt | 37 | 4.97 |
| gc | 90 | 12.10 |
| ag | 5 | 0.67 |
| aa | 5 | 0.67 |
| at | 18 | 2.42 |
| ac | 90 | 12.10 |
| tg | 86 | 11.56 |
| ta | 1 | 0.13 |
| cg | 51 | 6.85 |
| ca | 96 | 12.90 |
| ct | 32 | 4.30 |
| cc | 152 | 20.43 |
| g,c | 540 | 72.48 |
| a,t | 205 | 27.52 |
| CpG islands results:  **Results for 745 residue sequence "seqUPS29" starting "gcccctgccc"**  CpG island detected in region 9 to 208 (Obs/Exp = 0.63 and %GC = 73.00)  CpG island detected in region 10 to 209 (Obs/Exp = 0.64 and %GC = 72.50)  CpG island detected in region 11 to 210 (Obs/Exp = 0.64 and %GC = 72.50)  CpG island detected in region 12 to 211 (Obs/Exp = 0.64 and %GC = 72.50)  CpG island detected in region 13 to 212 (Obs/Exp = 0.63 and %GC = 73.00)  CpG island detected in region 14 to 213 (Obs/Exp = 0.63 and %GC = 73.00)  CpG island detected in region 15 to 214 (Obs/Exp = 0.63 and %GC = 73.00)  CpG island detected in region 16 to 215 (Obs/Exp = 0.63 and %GC = 73.00)  CpG island detected in region 17 to 216 (Obs/Exp = 0.67 and %GC = 73.00)  CpG island detected in region 18 to 217 (Obs/Exp = 0.63 and %GC = 73.00)  CpG island detected in region 19 to 218 (Obs/Exp = 0.63 and %GC = 73.00)  CpG island detected in region 20 to 219 (Obs/Exp = 0.62 and %GC = 73.50)  CpG island detected in region 21 to 220 (Obs/Exp = 0.63 and %GC = 73.00)  CpG island detected in region 22 to 221 (Obs/Exp = 0.63 and %GC = 72.50)  CpG island detected in region 23 to 222 (Obs/Exp = 0.62 and %GC = 73.00)  CpG island detected in region 24 to 223 (Obs/Exp = 0.62 and %GC = 73.00)  CpG island detected in region 25 to 224 (Obs/Exp = 0.61 and %GC = 73.50)  CpG island detected in region 26 to 225 (Obs/Exp = 0.61 and %GC = 73.50)  CpG island detected in region 27 to 226 (Obs/Exp = 0.61 and %GC = 74.00)  CpG island detected in region 28 to 227 (Obs/Exp = 0.61 and %GC = 74.00)  CpG island detected in region 29 to 228 (Obs/Exp = 0.61 and %GC = 74.00)  CpG island detected in region 53 to 252 (Obs/Exp = 0.61 and %GC = 72.50)  CpG island detected in region 54 to 253 (Obs/Exp = 0.61 and %GC = 72.50)  CpG island detected in region 55 to 254 (Obs/Exp = 0.64 and %GC = 72.50)  CpG island detected in region 56 to 255 (Obs/Exp = 0.61 and %GC = 72.00)  CpG island detected in region 57 to 256 (Obs/Exp = 0.61 and %GC = 72.00)  CpG island detected in region 63 to 262 (Obs/Exp = 0.61 and %GC = 72.00)  CpG island detected in region 64 to 263 (Obs/Exp = 0.60 and %GC = 72.50)  CpG island detected in region 65 to 264 (Obs/Exp = 0.60 and %GC = 72.50)  CpG island detected in region 68 to 267 (Obs/Exp = 0.60 and %GC = 72.00)  CpG island detected in region 99 to 298 (Obs/Exp = 0.61 and %GC = 72.50)  CpG island detected in region 100 to 299 (Obs/Exp = 0.61 and %GC = 72.50)  CpG island detected in region 101 to 300 (Obs/Exp = 0.64 and %GC = 72.50)  CpG island detected in region 102 to 301 (Obs/Exp = 0.61 and %GC = 72.00)  CpG island detected in region 103 to 302 (Obs/Exp = 0.61 and %GC = 72.00)  CpG island detected in region 104 to 303 (Obs/Exp = 0.60 and %GC = 72.50)  CpG island detected in region 105 to 304 (Obs/Exp = 0.61 and %GC = 72.50)  CpG island detected in region 106 to 305 (Obs/Exp = 0.61 and %GC = 72.00)  CpG island detected in region 107 to 306 (Obs/Exp = 0.61 and %GC = 72.00)  CpG island detected in region 109 to 308 (Obs/Exp = 0.61 and %GC = 72.00)  CpG island detected in region 110 to 309 (Obs/Exp = 0.60 and %GC = 72.50)  CpG island detected in region 111 to 310 (Obs/Exp = 0.60 and %GC = 72.50)  CpG island detected in region 114 to 313 (Obs/Exp = 0.60 and %GC = 72.00)  CpG island detected in region 461 to 660 (Obs/Exp = 0.61 and %GC = 72.00)  CpG island detected in region 462 to 661 (Obs/Exp = 0.61 and %GC = 72.00)  CpG island detected in region 463 to 662 (Obs/Exp = 0.61 and %GC = 72.00)  CpG island detected in region 464 to 663 (Obs/Exp = 0.61 and %GC = 72.00)  CpG island detected in region 465 to 664 (Obs/Exp = 0.61 and %GC = 72.00) | | |

Table S2. Results of search for nucleotide sequences that homologous to human UPS29 minisatellite in hominids (Ensembl, Blast/Blat search, <http://www.ensembl.org/Homo_sapiens/Tools/Blast?db=core;r=1:1297164-1297243;tl=8kfcxbeBe4SZPWNz-6219501-773577828>, accessed 07.06.2020).

| Ensemble, BLAST/BLAT search **Job name** Job 1: seq_UPS29 745 bp (human)  **Species Gorilla (*Gorilla gorilla gorilla*)** 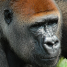  **Assembly** gorGor4  **Search type** BLASTN (NCBI Blast) | | | | | | | | | | | | | | |
| --- | --- | --- | --- | --- | --- | --- | --- | --- | --- | --- | --- | --- | --- | --- |
| **Genomic Location** | **Overlapping Gene(s)** | | **Orientation** | | **Query start** | | **Query end** | | | **Length** | | **Score** | **E-value** | **% ID** |
| [1:946408-946614](http://www.ensembl.org/Gorilla_gorilla/Location/View?r=1:946397-946624;tl=N3LVsKKEHxXtM9se-6276217-776269224) [[Sequence]](http://www.ensembl.org/Gorilla_gorilla/Tools/Blast/GenomicSeq?r=1:1297164-1297243;tl=N3LVsKKEHxXtM9se-6276217-776269224) | [ACAP3](http://www.ensembl.org/Gorilla_gorilla/Gene/Summary?db=core;g=ENSGGOG00000005348;r=1:1297164-1297243;tl=N3LVsKKEHxXtM9se-6276217-776269224) | | Reverse | | 541 | | 745 | | | 207 [[Sequence]](http://www.ensembl.org/Gorilla_gorilla/Tools/Blast/QuerySeq?r=1:1297164-1297243;tl=N3LVsKKEHxXtM9se-6276217-776269224) | | 338 | 3e-90 | 95.65 [[Alignment]](http://www.ensembl.org/Gorilla_gorilla/Tools/Blast/Alignment?r=1:1297164-1297243;tl=N3LVsKKEHxXtM9se-6276217-776269224) |
| [1:946422-946614](http://www.ensembl.org/Gorilla_gorilla/Location/View?r=1:946412-946623;tl=N3LVsKKEHxXtM9se-6276217-776269225) [[Sequence]](http://www.ensembl.org/Gorilla_gorilla/Tools/Blast/GenomicSeq?r=1:1297164-1297243;tl=N3LVsKKEHxXtM9se-6276217-776269225) | [ACAP3](http://www.ensembl.org/Gorilla_gorilla/Gene/Summary?db=core;g=ENSGGOG00000005348;r=1:1297164-1297243;tl=N3LVsKKEHxXtM9se-6276217-776269225) | | Reverse | | 315 | | 505 | | | 193 [[Sequence]](http://www.ensembl.org/Gorilla_gorilla/Tools/Blast/QuerySeq?r=1:1297164-1297243;tl=N3LVsKKEHxXtM9se-6276217-776269225) | | 287 | 8e-75 | 93.78 [[Alignment]](http://www.ensembl.org/Gorilla_gorilla/Tools/Blast/Alignment?r=1:1297164-1297243;tl=N3LVsKKEHxXtM9se-6276217-776269225) |
| [1:946422-946614](http://www.ensembl.org/Gorilla_gorilla/Location/View?r=1:946412-946623;tl=N3LVsKKEHxXtM9se-6276217-776269226) [[Sequence]](http://www.ensembl.org/Gorilla_gorilla/Tools/Blast/GenomicSeq?r=1:1297164-1297243;tl=N3LVsKKEHxXtM9se-6276217-776269226) | [ACAP3](http://www.ensembl.org/Gorilla_gorilla/Gene/Summary?db=core;g=ENSGGOG00000005348;r=1:1297164-1297243;tl=N3LVsKKEHxXtM9se-6276217-776269226) | | Reverse | | 1 | | 187 | | | 193 [[Sequence]](http://www.ensembl.org/Gorilla_gorilla/Tools/Blast/QuerySeq?r=1:1297164-1297243;tl=N3LVsKKEHxXtM9se-6276217-776269226) | | 287 | 8e-75 | 93.26 [[Alignment]](http://www.ensembl.org/Gorilla_gorilla/Tools/Blast/Alignment?r=1:1297164-1297243;tl=N3LVsKKEHxXtM9se-6276217-776269226) |
| [1:946422-946614](http://www.ensembl.org/Gorilla_gorilla/Location/View?r=1:946412-946623;tl=N3LVsKKEHxXtM9se-6276217-776269227) [[Sequence]](http://www.ensembl.org/Gorilla_gorilla/Tools/Blast/GenomicSeq?r=1:1297164-1297243;tl=N3LVsKKEHxXtM9se-6276217-776269227) | [ACAP3](http://www.ensembl.org/Gorilla_gorilla/Gene/Summary?db=core;g=ENSGGOG00000005348;r=1:1297164-1297243;tl=N3LVsKKEHxXtM9se-6276217-776269227) | | Reverse | | 449 | | 639 | | | 195 [[Sequence]](http://www.ensembl.org/Gorilla_gorilla/Tools/Blast/QuerySeq?r=1:1297164-1297243;tl=N3LVsKKEHxXtM9se-6276217-776269227) | | 271 | 5e-70 | 92.31 [[Alignment]](http://www.ensembl.org/Gorilla_gorilla/Tools/Blast/Alignment?r=1:1297164-1297243;tl=N3LVsKKEHxXtM9se-6276217-776269227) |
| [1:946422-946614](http://www.ensembl.org/Gorilla_gorilla/Location/View?r=1:946412-946623;tl=N3LVsKKEHxXtM9se-6276217-776269228) [[Sequence]](http://www.ensembl.org/Gorilla_gorilla/Tools/Blast/GenomicSeq?r=1:1297164-1297243;tl=N3LVsKKEHxXtM9se-6276217-776269228) | [ACAP3](http://www.ensembl.org/Gorilla_gorilla/Gene/Summary?db=core;g=ENSGGOG00000005348;r=1:1297164-1297243;tl=N3LVsKKEHxXtM9se-6276217-776269228) | | Reverse | | 223 | | 413 | | | 195 [[Sequence]](http://www.ensembl.org/Gorilla_gorilla/Tools/Blast/QuerySeq?r=1:1297164-1297243;tl=N3LVsKKEHxXtM9se-6276217-776269228) | | 271 | 5e-70 | 92.31 [[Alignment]](http://www.ensembl.org/Gorilla_gorilla/Tools/Blast/Alignment?r=1:1297164-1297243;tl=N3LVsKKEHxXtM9se-6276217-776269228) |
| [1:946422-946614](http://www.ensembl.org/Gorilla_gorilla/Location/View?r=1:946412-946623;tl=N3LVsKKEHxXtM9se-6276217-776269229) [[Sequence]](http://www.ensembl.org/Gorilla_gorilla/Tools/Blast/GenomicSeq?r=1:1297164-1297243;tl=N3LVsKKEHxXtM9se-6276217-776269229) | [ACAP3](http://www.ensembl.org/Gorilla_gorilla/Gene/Summary?db=core;g=ENSGGOG00000005348;r=1:1297164-1297243;tl=N3LVsKKEHxXtM9se-6276217-776269229) | | Reverse | | 91 | | 279 | | | 193 [[Sequence]](http://www.ensembl.org/Gorilla_gorilla/Tools/Blast/QuerySeq?r=1:1297164-1297243;tl=N3LVsKKEHxXtM9se-6276217-776269229) | | 259 | 2e-66 | 91.71 [[Alignment]](http://www.ensembl.org/Gorilla_gorilla/Tools/Blast/Alignment?r=1:1297164-1297243;tl=N3LVsKKEHxXtM9se-6276217-776269229) |
| [1:946408-946594](http://www.ensembl.org/Gorilla_gorilla/Location/View?r=1:946398-946603;tl=N3LVsKKEHxXtM9se-6276217-776269230) [[Sequence]](http://www.ensembl.org/Gorilla_gorilla/Tools/Blast/GenomicSeq?r=1:1297164-1297243;tl=N3LVsKKEHxXtM9se-6276217-776269230) | [ACAP3](http://www.ensembl.org/Gorilla_gorilla/Gene/Summary?db=core;g=ENSGGOG00000005348;r=1:1297164-1297243;tl=N3LVsKKEHxXtM9se-6276217-776269230) | | Reverse | | 379 | | 565 | | | 187 [[Sequence]](http://www.ensembl.org/Gorilla_gorilla/Tools/Blast/QuerySeq?r=1:1297164-1297243;tl=N3LVsKKEHxXtM9se-6276217-776269230) | | 251 | 4e-64 | 91.98 [[Alignment]](http://www.ensembl.org/Gorilla_gorilla/Tools/Blast/Alignment?r=1:1297164-1297243;tl=N3LVsKKEHxXtM9se-6276217-776269230) |
| [1:946408-946594](http://www.ensembl.org/Gorilla_gorilla/Location/View?r=1:946398-946603;tl=N3LVsKKEHxXtM9se-6276217-776269231) [[Sequence]](http://www.ensembl.org/Gorilla_gorilla/Tools/Blast/GenomicSeq?r=1:1297164-1297243;tl=N3LVsKKEHxXtM9se-6276217-776269231) | [ACAP3](http://www.ensembl.org/Gorilla_gorilla/Gene/Summary?db=core;g=ENSGGOG00000005348;r=1:1297164-1297243;tl=N3LVsKKEHxXtM9se-6276217-776269231) | | Reverse | | 153 | | 339 | | | 187 [[Sequence]](http://www.ensembl.org/Gorilla_gorilla/Tools/Blast/QuerySeq?r=1:1297164-1297243;tl=N3LVsKKEHxXtM9se-6276217-776269231) | | 251 | 4e-64 | 91.98 [[Alignment]](http://www.ensembl.org/Gorilla_gorilla/Tools/Blast/Alignment?r=1:1297164-1297243;tl=N3LVsKKEHxXtM9se-6276217-776269231) |
| [1:946422-946594](http://www.ensembl.org/Gorilla_gorilla/Location/View?r=1:946413-946602;tl=N3LVsKKEHxXtM9se-6276217-776269232) [[Sequence]](http://www.ensembl.org/Gorilla_gorilla/Tools/Blast/GenomicSeq?r=1:1297164-1297243;tl=N3LVsKKEHxXtM9se-6276217-776269232) | [ACAP3](http://www.ensembl.org/Gorilla_gorilla/Gene/Summary?db=core;g=ENSGGOG00000005348;r=1:1297164-1297243;tl=N3LVsKKEHxXtM9se-6276217-776269232) | | Reverse | | 425 | | 593 | | | 173 [[Sequence]](http://www.ensembl.org/Gorilla_gorilla/Tools/Blast/QuerySeq?r=1:1297164-1297243;tl=N3LVsKKEHxXtM9se-6276217-776269232) | | 223 | 9e-56 | 91.33 [[Alignment]](http://www.ensembl.org/Gorilla_gorilla/Tools/Blast/Alignment?r=1:1297164-1297243;tl=N3LVsKKEHxXtM9se-6276217-776269232) |
| [1:946422-946594](http://www.ensembl.org/Gorilla_gorilla/Location/View?r=1:946413-946602;tl=N3LVsKKEHxXtM9se-6276217-776269233) [[Sequence]](http://www.ensembl.org/Gorilla_gorilla/Tools/Blast/GenomicSeq?r=1:1297164-1297243;tl=N3LVsKKEHxXtM9se-6276217-776269233) | [ACAP3](http://www.ensembl.org/Gorilla_gorilla/Gene/Summary?db=core;g=ENSGGOG00000005348;r=1:1297164-1297243;tl=N3LVsKKEHxXtM9se-6276217-776269233) | | Reverse | | 199 | | 367 | | | 173 [[Sequence]](http://www.ensembl.org/Gorilla_gorilla/Tools/Blast/QuerySeq?r=1:1297164-1297243;tl=N3LVsKKEHxXtM9se-6276217-776269233) | | 223 | 9e-56 | 91.33 [[Alignment]](http://www.ensembl.org/Gorilla_gorilla/Tools/Blast/Alignment?r=1:1297164-1297243;tl=N3LVsKKEHxXtM9se-6276217-776269233) |
| [1:946422-946594](http://www.ensembl.org/Gorilla_gorilla/Location/View?r=1:946413-946602;tl=N3LVsKKEHxXtM9se-6276217-776269234) [[Sequence]](http://www.ensembl.org/Gorilla_gorilla/Tools/Blast/GenomicSeq?r=1:1297164-1297243;tl=N3LVsKKEHxXtM9se-6276217-776269234) | [ACAP3](http://www.ensembl.org/Gorilla_gorilla/Gene/Summary?db=core;g=ENSGGOG00000005348;r=1:1297164-1297243;tl=N3LVsKKEHxXtM9se-6276217-776269234) | | Reverse | | 517 | | 685 | | | 173 [[Sequence]](http://www.ensembl.org/Gorilla_gorilla/Tools/Blast/QuerySeq?r=1:1297164-1297243;tl=N3LVsKKEHxXtM9se-6276217-776269234) | | 219 | 1e-54 | 90.75 [[Alignment]](http://www.ensembl.org/Gorilla_gorilla/Tools/Blast/Alignment?r=1:1297164-1297243;tl=N3LVsKKEHxXtM9se-6276217-776269234) |
| [1:946408-946594](http://www.ensembl.org/Gorilla_gorilla/Location/View?r=1:946398-946603;tl=N3LVsKKEHxXtM9se-6276217-776269235) [[Sequence]](http://www.ensembl.org/Gorilla_gorilla/Tools/Blast/GenomicSeq?r=1:1297164-1297243;tl=N3LVsKKEHxXtM9se-6276217-776269235) | [ACAP3](http://www.ensembl.org/Gorilla_gorilla/Gene/Summary?db=core;g=ENSGGOG00000005348;r=1:1297164-1297243;tl=N3LVsKKEHxXtM9se-6276217-776269235) | | Reverse | | 67 | | 247 | | | 187 [[Sequence]](http://www.ensembl.org/Gorilla_gorilla/Tools/Blast/QuerySeq?r=1:1297164-1297243;tl=N3LVsKKEHxXtM9se-6276217-776269235) | | 211 | 3e-52 | 88.77 [[Alignment]](http://www.ensembl.org/Gorilla_gorilla/Tools/Blast/Alignment?r=1:1297164-1297243;tl=N3LVsKKEHxXtM9se-6276217-776269235) |
| [1:946408-946594](http://www.ensembl.org/Gorilla_gorilla/Location/View?r=1:946398-946603;tl=N3LVsKKEHxXtM9se-6276217-776269236) [[Sequence]](http://www.ensembl.org/Gorilla_gorilla/Tools/Blast/GenomicSeq?r=1:1297164-1297243;tl=N3LVsKKEHxXtM9se-6276217-776269236) | [ACAP3](http://www.ensembl.org/Gorilla_gorilla/Gene/Summary?db=core;g=ENSGGOG00000005348;r=1:1297164-1297243;tl=N3LVsKKEHxXtM9se-6276217-776269236) | | Reverse | | 291 | | 473 | | | 187 [[Sequence]](http://www.ensembl.org/Gorilla_gorilla/Tools/Blast/QuerySeq?r=1:1297164-1297243;tl=N3LVsKKEHxXtM9se-6276217-776269236) | | 208 | 5e-51 | 88.77 [[Alignment]](http://www.ensembl.org/Gorilla_gorilla/Tools/Blast/Alignment?r=1:1297164-1297243;tl=N3LVsKKEHxXtM9se-6276217-776269236) |
| [1:946468-946594](http://www.ensembl.org/Gorilla_gorilla/Location/View?r=1:946461-946600;tl=N3LVsKKEHxXtM9se-6276217-776269237) [[Sequence]](http://www.ensembl.org/Gorilla_gorilla/Tools/Blast/GenomicSeq?r=1:1297164-1297243;tl=N3LVsKKEHxXtM9se-6276217-776269237) | [ACAP3](http://www.ensembl.org/Gorilla_gorilla/Gene/Summary?db=core;g=ENSGGOG00000005348;r=1:1297164-1297243;tl=N3LVsKKEHxXtM9se-6276217-776269237) | | Reverse | | 605 | | 731 | | | 127 [[Sequence]](http://www.ensembl.org/Gorilla_gorilla/Tools/Blast/QuerySeq?r=1:1297164-1297243;tl=N3LVsKKEHxXtM9se-6276217-776269237) | | 180 | 1e-42 | 92.91 [[Alignment]](http://www.ensembl.org/Gorilla_gorilla/Tools/Blast/Alignment?r=1:1297164-1297243;tl=N3LVsKKEHxXtM9se-6276217-776269237) |
| [1:946422-946570](http://www.ensembl.org/Gorilla_gorilla/Location/View?r=1:946414-946577;tl=N3LVsKKEHxXtM9se-6276217-776269238) [[Sequence]](http://www.ensembl.org/Gorilla_gorilla/Tools/Blast/GenomicSeq?r=1:1297164-1297243;tl=N3LVsKKEHxXtM9se-6276217-776269238) | [ACAP3](http://www.ensembl.org/Gorilla_gorilla/Gene/Summary?db=core;g=ENSGGOG00000005348;r=1:1297164-1297243;tl=N3LVsKKEHxXtM9se-6276217-776269238) | | Reverse | | 1 | | 141 | | | 149 [[Sequence]](http://www.ensembl.org/Gorilla_gorilla/Tools/Blast/QuerySeq?r=1:1297164-1297243;tl=N3LVsKKEHxXtM9se-6276217-776269238) | | 168 | 4e-39 | 88.59 [[Alignment]](http://www.ensembl.org/Gorilla_gorilla/Tools/Blast/Alignment?r=1:1297164-1297243;tl=N3LVsKKEHxXtM9se-6276217-776269238) |
| [1:946422-946524](http://www.ensembl.org/Gorilla_gorilla/Location/View?r=1:946416-946529;tl=N3LVsKKEHxXtM9se-6276217-776269239) [[Sequence]](http://www.ensembl.org/Gorilla_gorilla/Tools/Blast/GenomicSeq?r=1:1297164-1297243;tl=N3LVsKKEHxXtM9se-6276217-776269239) | [ACAP3](http://www.ensembl.org/Gorilla_gorilla/Gene/Summary?db=core;g=ENSGGOG00000005348;r=1:1297164-1297243;tl=N3LVsKKEHxXtM9se-6276217-776269239) | | Reverse | | 1 | | 101 | | | 103 [[Sequence]](http://www.ensembl.org/Gorilla_gorilla/Tools/Blast/QuerySeq?r=1:1297164-1297243;tl=N3LVsKKEHxXtM9se-6276217-776269239) | | 125 | 5e-26 | 90.29 [[Alignment]](http://www.ensembl.org/Gorilla_gorilla/Tools/Blast/Alignment?r=1:1297164-1297243;tl=N3LVsKKEHxXtM9se-6276217-776269239) |
| [1:946514-946594](http://www.ensembl.org/Gorilla_gorilla/Location/View?r=1:946510-946598;tl=N3LVsKKEHxXtM9se-6276217-776269240) [[Sequence]](http://www.ensembl.org/Gorilla_gorilla/Tools/Blast/GenomicSeq?r=1:1297164-1297243;tl=N3LVsKKEHxXtM9se-6276217-776269240) | [ACAP3](http://www.ensembl.org/Gorilla_gorilla/Gene/Summary?db=core;g=ENSGGOG00000005348;r=1:1297164-1297243;tl=N3LVsKKEHxXtM9se-6276217-776269240) | | Reverse | | 651 | | 731 | | | 81 [[Sequence]](http://www.ensembl.org/Gorilla_gorilla/Tools/Blast/QuerySeq?r=1:1297164-1297243;tl=N3LVsKKEHxXtM9se-6276217-776269240) | | 97.4 | 1e-17 | 90.12 [[Alignment]](http://www.ensembl.org/Gorilla_gorilla/Tools/Blast/Alignment?r=1:1297164-1297243;tl=N3LVsKKEHxXtM9se-6276217-776269240) |
| [1:946422-946478](http://www.ensembl.org/Gorilla_gorilla/Location/View?r=1:946419-946480;tl=N3LVsKKEHxXtM9se-6276217-776269241) [[Sequence]](http://www.ensembl.org/Gorilla_gorilla/Tools/Blast/GenomicSeq?r=1:1297164-1297243;tl=N3LVsKKEHxXtM9se-6276217-776269241) | [ACAP3](http://www.ensembl.org/Gorilla_gorilla/Gene/Summary?db=core;g=ENSGGOG00000005348;r=1:1297164-1297243;tl=N3LVsKKEHxXtM9se-6276217-776269241) | | Reverse | | 1 | | 55 | | | 57 [[Sequence]](http://www.ensembl.org/Gorilla_gorilla/Tools/Blast/QuerySeq?r=1:1297164-1297243;tl=N3LVsKKEHxXtM9se-6276217-776269241) | | 65.7 | 4e-08 | 89.47 [[Alignment]](http://www.ensembl.org/Gorilla_gorilla/Tools/Blast/Alignment?r=1:1297164-1297243;tl=N3LVsKKEHxXtM9se-6276217-776269241) |
| [1:946560-946614](http://www.ensembl.org/Gorilla_gorilla/Location/View?r=1:946557-946616;tl=N3LVsKKEHxXtM9se-6276217-776269242) [[Sequence]](http://www.ensembl.org/Gorilla_gorilla/Tools/Blast/GenomicSeq?r=1:1297164-1297243;tl=N3LVsKKEHxXtM9se-6276217-776269242) | [ACAP3](http://www.ensembl.org/Gorilla_gorilla/Gene/Summary?db=core;g=ENSGGOG00000005348;r=1:1297164-1297243;tl=N3LVsKKEHxXtM9se-6276217-776269242) | | Reverse | | 675 | | 731 | | | 57 [[Sequence]](http://www.ensembl.org/Gorilla_gorilla/Tools/Blast/QuerySeq?r=1:1297164-1297243;tl=N3LVsKKEHxXtM9se-6276217-776269242) | | 49.9 | 0.002 | 85.96 [[Alignment]](http://www.ensembl.org/Gorilla_gorilla/Tools/Blast/Alignment?r=1:1297164-1297243;tl=N3LVsKKEHxXtM9se-6276217-776269242) |
| BLAST/BLAT Alignment **BLAST/BLAT type** BLASTN  **Query location** seq_UPS29 541 to 745 (+)  **Database location** 1: 946408 to 946614 (-)  **Genomic location** 1: 946408 to 946614 (-)  **Alignment score** 338  **E-value** 3e-90  **Alignment length** 207  **Percentage identity** 95.65  seq_UPS29: 541 GCCCCTGCCCCAAGCCCGTGCACACA--TGTGCCACCGGGGATGGCCCCTGCCCCGCGCC seq_UPS29: 600  1 \|\|\|\|\|\|\|\|\|\|\|\|\|\|\|\|\| \|\|\|\|\|\|\|\| \|\|\|\|\|\|\|\|\|\|\|\|\|\|\|\|\|\|\|\|\|\|\|\|\|\|\|\|\|\|\|\| 60  1:946614 GCCCCTGCCCCAAGCCCCTGCACACACATGTGCCACCGGGGATGGCCCCTGCCCCGCGCC 1:946555  seq_UPS29: 601 TGTGCACACACACACGTGCCACTGGGGATGGCCCCTGCCCCGCGCCTGTGCACACACACA seq_UPS29: 660  61 \|\|\|\|\|\|\|\|\|\|\|\|\|\|\|\|\|\|\|\|\|\| \|\|\|\|\|\|\|\|\|\|\|\| \|\|\|\|\|\| \|\|\|\|\|\|\|\|\|\|\|\|\|\|\|\|\| 120  1:946554 TGTGCACACACACACGTGCCACCGGGGATGGCCCCCGCCCCGTGCCTGTGCACACACACA 1:946495  seq_UPS29: 661 CGTGCCACTGGGGATGGCCCCTGCCCCGTGCCGGTGCACACACCTACGTGCCACTGGGGA seq_UPS29: 720  121 \|\|\|\|\|\|\| \|\|\|\|\|\|\|\|\|\|\|\|\|\|\|\|\|\|\| \|\|\|\|\|\|\|\|\|\|\|\|\|\|\|\|\|\|\|\|\|\|\|\|\|\|\|\|\|\|\| 180  1:946494 TGTGCCACCGGGGATGGCCCCTGCCCCGCGCCGGTGCACACACCTACGTGCCACTGGGGA 1:946435  seq_UPS29: 721 TGGCCCCTGCCCCACACACGTGCACAC seq_UPS29: 747  181 \|\|\|\|\|\|\|\|\|\|\|\|\|\|\|\|\|\|\|\|\|\|\|\|\|\|\| 207  1:946434 TGGCCCCTGCCCCACACACGTGCACAC 1:946406  **BLAST/BLAT type** BLASTN  **Query location** seq_UPS29 449 to 639 (+)  **Database location** 1: 946422 to 946614 (-)  **Genomic location** 1: 946422 to 946614 (-)  **Alignment score** 271  **E-value** 5e-70  **Alignment length** 195  **Percentage identity** 92.31  seq_UPS29: 449 GCCCCTGCCCCAAGCCCGTGCACACACACACGTGCCACCGGGGATGGCCCCTGCCCCGCG seq_UPS29: 508  1 \|\|\|\|\|\|\|\|\|\|\|\|\|\|\|\|\| \|\|\|\|\|\|\|\|\|\| \|\|\|\|\|\|\|\|\|\|\|\|\|\|\|\|\|\|\|\|\|\|\|\|\|\|\|\|\| 60  1:946614 GCCCCTGCCCCAAGCCCCTGCACACACA--TGTGCCACCGGGGATGGCCCCTGCCCCGCG 1:946555  seq_UPS29: 509 CCTGTGCACACACACACGTGCCACCGGGGATGGCCCCTGCCCCAAGCCCGTGCACACA-- seq_UPS29: 568  61 \|\|\|\|\|\|\|\|\|\|\|\|\|\|\|\|\|\|\|\|\|\|\|\|\|\|\|\|\|\|\|\|\|\|\|\|\| \|\|\|\|\| \|\|\| \|\|\|\|\|\|\|\|\| 120  1:946554 CCTGTGCACACACACACGTGCCACCGGGGATGGCCCCCGCCCCGTGCCTGTGCACACACA 1:946495  seq_UPS29: 569 --TGTGCCACCGGGGATGGCCCCTGCCCCGCGCCTGTGCACACACACACGTGCCACTGGG seq_UPS29: 628  121 \|\|\|\|\|\|\|\|\|\|\|\|\|\|\|\|\|\|\|\|\|\|\|\|\|\|\|\|\|\|\|\| \|\|\|\|\|\|\|\|\|\| \|\|\|\|\|\|\|\|\|\|\|\|\| 180  1:946494 CATGTGCCACCGGGGATGGCCCCTGCCCCGCGCCGGTGCACACACCTACGTGCCACTGGG 1:946435  seq_UPS29: 629 GATGGCCCCTGCCCC seq_UPS29: 643  181 \|\|\|\|\|\|\|\|\|\|\|\|\|\|\| 195  1:946434 GATGGCCCCTGCCCC 1:946418 | | | | | | | | | | | | | | |
| Ensemble, BLAST/BLAT search **Job name** Job 2: seq_UPS29 745 bp (human)  **Species Bonobo (*Pan paniscus*)** 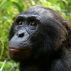  **Assembly** panpan1.1  **Search type** BLASTN (NCBI Blast) | | | | | | | | | | | | | | |
| **Genomic Location** | | **Overlapping Gene(s)** | **Orientation** | **Query start** | | **Query end** | | | **Length** | | **Score** | | **E-value** | **%ID** |
| [1:1253084-1253338](http://www.ensembl.org/Pan_paniscus/Location/View?r=1:1253071-1253350;tl=N3LVsKKEHxXtM9se-6276218-776269276) [[Sequence]](http://www.ensembl.org/Pan_paniscus/Tools/Blast/GenomicSeq?r=1:1297164-1297243;tl=N3LVsKKEHxXtM9se-6276218-776269276) | | [ACAP3](http://www.ensembl.org/Pan_paniscus/Gene/Summary?db=core;g=ENSPPAG00000035731;r=1:1297164-1297243;tl=N3LVsKKEHxXtM9se-6276218-776269276) | Reverse | 495 | | 745 | | | 255 [[Sequence]](http://www.ensembl.org/Pan_paniscus/Tools/Blast/QuerySeq?r=1:1297164-1297243;tl=N3LVsKKEHxXtM9se-6276218-776269276) | | 381 | | 2e-103 | 93.73 [[Alignment]](http://www.ensembl.org/Pan_paniscus/Tools/Blast/Alignment?r=1:1297164-1297243;tl=N3LVsKKEHxXtM9se-6276218-776269276) |
| [1:1253084-1253338](http://www.ensembl.org/Pan_paniscus/Location/View?r=1:1253071-1253350;tl=N3LVsKKEHxXtM9se-6276218-776269277) [[Sequence]](http://www.ensembl.org/Pan_paniscus/Tools/Blast/GenomicSeq?r=1:1297164-1297243;tl=N3LVsKKEHxXtM9se-6276218-776269277) | | [ACAP3](http://www.ensembl.org/Pan_paniscus/Gene/Summary?db=core;g=ENSPPAG00000035731;r=1:1297164-1297243;tl=N3LVsKKEHxXtM9se-6276218-776269277) | Reverse | 223 | | 473 | | | 255 [[Sequence]](http://www.ensembl.org/Pan_paniscus/Tools/Blast/QuerySeq?r=1:1297164-1297243;tl=N3LVsKKEHxXtM9se-6276218-776269277) | | 350 | | 8e-94 | 92.16 [[Alignment]](http://www.ensembl.org/Pan_paniscus/Tools/Blast/Alignment?r=1:1297164-1297243;tl=N3LVsKKEHxXtM9se-6276218-776269277) |
| [1:1253098-1253338](http://www.ensembl.org/Pan_paniscus/Location/View?r=1:1253086-1253350;tl=N3LVsKKEHxXtM9se-6276218-776269278) [[Sequence]](http://www.ensembl.org/Pan_paniscus/Tools/Blast/GenomicSeq?r=1:1297164-1297243;tl=N3LVsKKEHxXtM9se-6276218-776269278) | | [ACAP3](http://www.ensembl.org/Pan_paniscus/Gene/Summary?db=core;g=ENSPPAG00000035731;r=1:1297164-1297243;tl=N3LVsKKEHxXtM9se-6276218-776269278) | Reverse | 357 | | 593 | | | 241 [[Sequence]](http://www.ensembl.org/Pan_paniscus/Tools/Blast/QuerySeq?r=1:1297164-1297243;tl=N3LVsKKEHxXtM9se-6276218-776269278) | | 350 | | 8e-94 | 93.36 [[Alignment]](http://www.ensembl.org/Pan_paniscus/Tools/Blast/Alignment?r=1:1297164-1297243;tl=N3LVsKKEHxXtM9se-6276218-776269278) |
| [1:1253098-1253338](http://www.ensembl.org/Pan_paniscus/Location/View?r=1:1253086-1253350;tl=N3LVsKKEHxXtM9se-6276218-776269279) [[Sequence]](http://www.ensembl.org/Pan_paniscus/Tools/Blast/GenomicSeq?r=1:1297164-1297243;tl=N3LVsKKEHxXtM9se-6276218-776269279) | | [ACAP3](http://www.ensembl.org/Pan_paniscus/Gene/Summary?db=core;g=ENSPPAG00000035731;r=1:1297164-1297243;tl=N3LVsKKEHxXtM9se-6276218-776269279) | Reverse | 131 | | 367 | | | 241 [[Sequence]](http://www.ensembl.org/Pan_paniscus/Tools/Blast/QuerySeq?r=1:1297164-1297243;tl=N3LVsKKEHxXtM9se-6276218-776269279) | | 350 | | 8e-94 | 93.36 [[Alignment]](http://www.ensembl.org/Pan_paniscus/Tools/Blast/Alignment?r=1:1297164-1297243;tl=N3LVsKKEHxXtM9se-6276218-776269279) |
| [1:1253098-1253338](http://www.ensembl.org/Pan_paniscus/Location/View?r=1:1253086-1253350;tl=N3LVsKKEHxXtM9se-6276218-776269280) [[Sequence]](http://www.ensembl.org/Pan_paniscus/Tools/Blast/GenomicSeq?r=1:1297164-1297243;tl=N3LVsKKEHxXtM9se-6276218-776269280) | | [ACAP3](http://www.ensembl.org/Pan_paniscus/Gene/Summary?db=core;g=ENSPPAG00000035731;r=1:1297164-1297243;tl=N3LVsKKEHxXtM9se-6276218-776269280) | Reverse | 449 | | 685 | | | 241 [[Sequence]](http://www.ensembl.org/Pan_paniscus/Tools/Blast/QuerySeq?r=1:1297164-1297243;tl=N3LVsKKEHxXtM9se-6276218-776269280) | | 346 | | 1e-92 | 92.95 [[Alignment]](http://www.ensembl.org/Pan_paniscus/Tools/Blast/Alignment?r=1:1297164-1297243;tl=N3LVsKKEHxXtM9se-6276218-776269280) |
| [1:1253084-1253338](http://www.ensembl.org/Pan_paniscus/Location/View?r=1:1253071-1253350;tl=N3LVsKKEHxXtM9se-6276218-776269281) [[Sequence]](http://www.ensembl.org/Pan_paniscus/Tools/Blast/GenomicSeq?r=1:1297164-1297243;tl=N3LVsKKEHxXtM9se-6276218-776269281) | | [ACAP3](http://www.ensembl.org/Pan_paniscus/Gene/Summary?db=core;g=ENSPPAG00000035731;r=1:1297164-1297243;tl=N3LVsKKEHxXtM9se-6276218-776269281) | Reverse | 315 | | 565 | | | 255 [[Sequence]](http://www.ensembl.org/Pan_paniscus/Tools/Blast/QuerySeq?r=1:1297164-1297243;tl=N3LVsKKEHxXtM9se-6276218-776269281) | | 334 | | 5e-89 | 91.37 [[Alignment]](http://www.ensembl.org/Pan_paniscus/Tools/Blast/Alignment?r=1:1297164-1297243;tl=N3LVsKKEHxXtM9se-6276218-776269281) |
| [1:1253084-1253312](http://www.ensembl.org/Pan_paniscus/Location/View?r=1:1253072-1253323;tl=N3LVsKKEHxXtM9se-6276218-776269282) [[Sequence]](http://www.ensembl.org/Pan_paniscus/Tools/Blast/GenomicSeq?r=1:1297164-1297243;tl=N3LVsKKEHxXtM9se-6276218-776269282) | | [ACAP3](http://www.ensembl.org/Pan_paniscus/Gene/Summary?db=core;g=ENSPPAG00000035731;r=1:1297164-1297243;tl=N3LVsKKEHxXtM9se-6276218-776269282) | Reverse | 111 | | 339 | | | 229 [[Sequence]](http://www.ensembl.org/Pan_paniscus/Tools/Blast/QuerySeq?r=1:1297164-1297243;tl=N3LVsKKEHxXtM9se-6276218-776269282) | | 334 | | 5e-89 | 93.45 [[Alignment]](http://www.ensembl.org/Pan_paniscus/Tools/Blast/Alignment?r=1:1297164-1297243;tl=N3LVsKKEHxXtM9se-6276218-776269282) |
| [1:1253098-1253338](http://www.ensembl.org/Pan_paniscus/Location/View?r=1:1253086-1253350;tl=N3LVsKKEHxXtM9se-6276218-776269283) [[Sequence]](http://www.ensembl.org/Pan_paniscus/Tools/Blast/GenomicSeq?r=1:1297164-1297243;tl=N3LVsKKEHxXtM9se-6276218-776269283) | | [ACAP3](http://www.ensembl.org/Pan_paniscus/Gene/Summary?db=core;g=ENSPPAG00000035731;r=1:1297164-1297243;tl=N3LVsKKEHxXtM9se-6276218-776269283) | Reverse | 403 | | 639 | | | 241 [[Sequence]](http://www.ensembl.org/Pan_paniscus/Tools/Blast/QuerySeq?r=1:1297164-1297243;tl=N3LVsKKEHxXtM9se-6276218-776269283) | | 330 | | 7e-88 | 92.12 [[Alignment]](http://www.ensembl.org/Pan_paniscus/Tools/Blast/Alignment?r=1:1297164-1297243;tl=N3LVsKKEHxXtM9se-6276218-776269283) |
| [1:1253098-1253338](http://www.ensembl.org/Pan_paniscus/Location/View?r=1:1253086-1253350;tl=N3LVsKKEHxXtM9se-6276218-776269284) [[Sequence]](http://www.ensembl.org/Pan_paniscus/Tools/Blast/GenomicSeq?r=1:1297164-1297243;tl=N3LVsKKEHxXtM9se-6276218-776269284) | | [ACAP3](http://www.ensembl.org/Pan_paniscus/Gene/Summary?db=core;g=ENSPPAG00000035731;r=1:1297164-1297243;tl=N3LVsKKEHxXtM9se-6276218-776269284) | Reverse | 177 | | 413 | | | 241 [[Sequence]](http://www.ensembl.org/Pan_paniscus/Tools/Blast/QuerySeq?r=1:1297164-1297243;tl=N3LVsKKEHxXtM9se-6276218-776269284) | | 330 | | 7e-88 | 92.12 [[Alignment]](http://www.ensembl.org/Pan_paniscus/Tools/Blast/Alignment?r=1:1297164-1297243;tl=N3LVsKKEHxXtM9se-6276218-776269284) |
| [1:1253084-1253338](http://www.ensembl.org/Pan_paniscus/Location/View?r=1:1253071-1253350;tl=N3LVsKKEHxXtM9se-6276218-776269285) [[Sequence]](http://www.ensembl.org/Pan_paniscus/Tools/Blast/GenomicSeq?r=1:1297164-1297243;tl=N3LVsKKEHxXtM9se-6276218-776269285) | | [ACAP3](http://www.ensembl.org/Pan_paniscus/Gene/Summary?db=core;g=ENSPPAG00000035731;r=1:1297164-1297243;tl=N3LVsKKEHxXtM9se-6276218-776269285) | Reverse | 1 | | 247 | | | 255 [[Sequence]](http://www.ensembl.org/Pan_paniscus/Tools/Blast/QuerySeq?r=1:1297164-1297243;tl=N3LVsKKEHxXtM9se-6276218-776269285) | | 322 | | 2e-85 | 90.59 [[Alignment]](http://www.ensembl.org/Pan_paniscus/Tools/Blast/Alignment?r=1:1297164-1297243;tl=N3LVsKKEHxXtM9se-6276218-776269285) |
| [1:1253098-1253338](http://www.ensembl.org/Pan_paniscus/Location/View?r=1:1253086-1253350;tl=N3LVsKKEHxXtM9se-6276218-776269286) [[Sequence]](http://www.ensembl.org/Pan_paniscus/Tools/Blast/GenomicSeq?r=1:1297164-1297243;tl=N3LVsKKEHxXtM9se-6276218-776269286) | | [ACAP3](http://www.ensembl.org/Pan_paniscus/Gene/Summary?db=core;g=ENSPPAG00000035731;r=1:1297164-1297243;tl=N3LVsKKEHxXtM9se-6276218-776269286) | Reverse | 45 | | 279 | | | 241 [[Sequence]](http://www.ensembl.org/Pan_paniscus/Tools/Blast/QuerySeq?r=1:1297164-1297243;tl=N3LVsKKEHxXtM9se-6276218-776269286) | | 318 | | 3e-84 | 91.29 [[Alignment]](http://www.ensembl.org/Pan_paniscus/Tools/Blast/Alignment?r=1:1297164-1297243;tl=N3LVsKKEHxXtM9se-6276218-776269286) |
| [1:1253098-1253338](http://www.ensembl.org/Pan_paniscus/Location/View?r=1:1253086-1253350;tl=N3LVsKKEHxXtM9se-6276218-776269287) [[Sequence]](http://www.ensembl.org/Pan_paniscus/Tools/Blast/GenomicSeq?r=1:1297164-1297243;tl=N3LVsKKEHxXtM9se-6276218-776269287) | | [ACAP3](http://www.ensembl.org/Pan_paniscus/Gene/Summary?db=core;g=ENSPPAG00000035731;r=1:1297164-1297243;tl=N3LVsKKEHxXtM9se-6276218-776269287) | Reverse | 269 | | 505 | | | 241 [[Sequence]](http://www.ensembl.org/Pan_paniscus/Tools/Blast/QuerySeq?r=1:1297164-1297243;tl=N3LVsKKEHxXtM9se-6276218-776269287) | | 306 | | 1e-80 | 90.87 [[Alignment]](http://www.ensembl.org/Pan_paniscus/Tools/Blast/Alignment?r=1:1297164-1297243;tl=N3LVsKKEHxXtM9se-6276218-776269287) |
| [1:1253144-1253338](http://www.ensembl.org/Pan_paniscus/Location/View?r=1:1253134-1253347;tl=N3LVsKKEHxXtM9se-6276218-776269288) [[Sequence]](http://www.ensembl.org/Pan_paniscus/Tools/Blast/GenomicSeq?r=1:1297164-1297243;tl=N3LVsKKEHxXtM9se-6276218-776269288) | | [ACAP3](http://www.ensembl.org/Pan_paniscus/Gene/Summary?db=core;g=ENSPPAG00000035731;r=1:1297164-1297243;tl=N3LVsKKEHxXtM9se-6276218-776269288) | Reverse | 541 | | 731 | | | 195 [[Sequence]](http://www.ensembl.org/Pan_paniscus/Tools/Blast/QuerySeq?r=1:1297164-1297243;tl=N3LVsKKEHxXtM9se-6276218-776269288) | | 247 | | 7e-63 | 90.77 [[Alignment]](http://www.ensembl.org/Pan_paniscus/Tools/Blast/Alignment?r=1:1297164-1297243;tl=N3LVsKKEHxXtM9se-6276218-776269288) |
| [1:1253098-1253270](http://www.ensembl.org/Pan_paniscus/Location/View?r=1:1253089-1253278;tl=N3LVsKKEHxXtM9se-6276218-776269289) [[Sequence]](http://www.ensembl.org/Pan_paniscus/Tools/Blast/GenomicSeq?r=1:1297164-1297243;tl=N3LVsKKEHxXtM9se-6276218-776269289) | | [ACAP3](http://www.ensembl.org/Pan_paniscus/Gene/Summary?db=core;g=ENSPPAG00000035731;r=1:1297164-1297243;tl=N3LVsKKEHxXtM9se-6276218-776269289) | Reverse | 21 | | 187 | | | 173 [[Sequence]](http://www.ensembl.org/Pan_paniscus/Tools/Blast/QuerySeq?r=1:1297164-1297243;tl=N3LVsKKEHxXtM9se-6276218-776269289) | | 239 | | 2e-60 | 91.91 [[Alignment]](http://www.ensembl.org/Pan_paniscus/Tools/Blast/Alignment?r=1:1297164-1297243;tl=N3LVsKKEHxXtM9se-6276218-776269289) |
| [1:1253098-1253246](http://www.ensembl.org/Pan_paniscus/Location/View?r=1:1253090-1253253;tl=N3LVsKKEHxXtM9se-6276218-776269290) [[Sequence]](http://www.ensembl.org/Pan_paniscus/Tools/Blast/GenomicSeq?r=1:1297164-1297243;tl=N3LVsKKEHxXtM9se-6276218-776269290) | | [ACAP3](http://www.ensembl.org/Pan_paniscus/Gene/Summary?db=core;g=ENSPPAG00000035731;r=1:1297164-1297243;tl=N3LVsKKEHxXtM9se-6276218-776269290) | Reverse | 1 | | 141 | | | 149 [[Sequence]](http://www.ensembl.org/Pan_paniscus/Tools/Blast/QuerySeq?r=1:1297164-1297243;tl=N3LVsKKEHxXtM9se-6276218-776269290) | | 200 | | 1e-48 | 91.28 [[Alignment]](http://www.ensembl.org/Pan_paniscus/Tools/Blast/Alignment?r=1:1297164-1297243;tl=N3LVsKKEHxXtM9se-6276218-776269290) |
| [1:1253188-1253338](http://www.ensembl.org/Pan_paniscus/Location/View?r=1:1253180-1253345;tl=N3LVsKKEHxXtM9se-6276218-776269291) [[Sequence]](http://www.ensembl.org/Pan_paniscus/Tools/Blast/GenomicSeq?r=1:1297164-1297243;tl=N3LVsKKEHxXtM9se-6276218-776269291) | | [ACAP3](http://www.ensembl.org/Pan_paniscus/Gene/Summary?db=core;g=ENSPPAG00000035731;r=1:1297164-1297243;tl=N3LVsKKEHxXtM9se-6276218-776269291) | Reverse | 583 | | 733 | | | 151 [[Sequence]](http://www.ensembl.org/Pan_paniscus/Tools/Blast/QuerySeq?r=1:1297164-1297243;tl=N3LVsKKEHxXtM9se-6276218-776269291) | | 196 | | 2e-47 | 91.39 [[Alignment]](http://www.ensembl.org/Pan_paniscus/Tools/Blast/Alignment?r=1:1297164-1297243;tl=N3LVsKKEHxXtM9se-6276218-776269291) |
| [1:1253234-1253338](http://www.ensembl.org/Pan_paniscus/Location/View?r=1:1253228-1253343;tl=N3LVsKKEHxXtM9se-6276218-776269292) [[Sequence]](http://www.ensembl.org/Pan_paniscus/Tools/Blast/GenomicSeq?r=1:1297164-1297243;tl=N3LVsKKEHxXtM9se-6276218-776269292) | | [ACAP3](http://www.ensembl.org/Pan_paniscus/Gene/Summary?db=core;g=ENSPPAG00000035731;r=1:1297164-1297243;tl=N3LVsKKEHxXtM9se-6276218-776269292) | Reverse | 629 | | 733 | | | 105 [[Sequence]](http://www.ensembl.org/Pan_paniscus/Tools/Blast/QuerySeq?r=1:1297164-1297243;tl=N3LVsKKEHxXtM9se-6276218-776269292) | | 136 | | 1e-29 | 91.43 [[Alignment]](http://www.ensembl.org/Pan_paniscus/Tools/Blast/Alignment?r=1:1297164-1297243;tl=N3LVsKKEHxXtM9se-6276218-776269292) |
| [1:1253098-1253200](http://www.ensembl.org/Pan_paniscus/Location/View?r=1:1253092-1253205;tl=N3LVsKKEHxXtM9se-6276218-776269293) [[Sequence]](http://www.ensembl.org/Pan_paniscus/Tools/Blast/GenomicSeq?r=1:1297164-1297243;tl=N3LVsKKEHxXtM9se-6276218-776269293) | | [ACAP3](http://www.ensembl.org/Pan_paniscus/Gene/Summary?db=core;g=ENSPPAG00000035731;r=1:1297164-1297243;tl=N3LVsKKEHxXtM9se-6276218-776269293) | Reverse | 1 | | 101 | | | 103 [[Sequence]](http://www.ensembl.org/Pan_paniscus/Tools/Blast/QuerySeq?r=1:1297164-1297243;tl=N3LVsKKEHxXtM9se-6276218-776269293) | | 132 | | 2e-28 | 91.26 [[Alignment]](http://www.ensembl.org/Pan_paniscus/Tools/Blast/Alignment?r=1:1297164-1297243;tl=N3LVsKKEHxXtM9se-6276218-776269293) |
| [1:1253098-1253154](http://www.ensembl.org/Pan_paniscus/Location/View?r=1:1253095-1253156;tl=N3LVsKKEHxXtM9se-6276218-776269294) [[Sequence]](http://www.ensembl.org/Pan_paniscus/Tools/Blast/GenomicSeq?r=1:1297164-1297243;tl=N3LVsKKEHxXtM9se-6276218-776269294) | | [ACAP3](http://www.ensembl.org/Pan_paniscus/Gene/Summary?db=core;g=ENSPPAG00000035731;r=1:1297164-1297243;tl=N3LVsKKEHxXtM9se-6276218-776269294) | Reverse | 1 | | 55 | | | 57 [[Sequence]](http://www.ensembl.org/Pan_paniscus/Tools/Blast/QuerySeq?r=1:1297164-1297243;tl=N3LVsKKEHxXtM9se-6276218-776269294) | | 65.7 | | 4e-08 | 89.47 [[Alignment]](http://www.ensembl.org/Pan_paniscus/Tools/Blast/Alignment?r=1:1297164-1297243;tl=N3LVsKKEHxXtM9se-6276218-776269294) |
| [1:1253282-1253338](http://www.ensembl.org/Pan_paniscus/Location/View?r=1:1253279-1253340;tl=N3LVsKKEHxXtM9se-6276218-776269295) [[Sequence]](http://www.ensembl.org/Pan_paniscus/Tools/Blast/GenomicSeq?r=1:1297164-1297243;tl=N3LVsKKEHxXtM9se-6276218-776269295) | | [ACAP3](http://www.ensembl.org/Pan_paniscus/Gene/Summary?db=core;g=ENSPPAG00000035731;r=1:1297164-1297243;tl=N3LVsKKEHxXtM9se-6276218-776269295) | Reverse | 675 | | 731 | | | 57 [[Sequence]](http://www.ensembl.org/Pan_paniscus/Tools/Blast/QuerySeq?r=1:1297164-1297243;tl=N3LVsKKEHxXtM9se-6276218-776269295) | | 49.9 | | 0.002 | 85.96 [[Alignment]](http://www.ensembl.org/Pan_paniscus/Tools/Blast/Alignment?r=1:1297164-1297243;tl=N3LVsKKEHxXtM9se-6276218-776269295) |
| BLAST/BLAT Alignment **BLAST/BLAT type** BLASTN  **Query location** seq_UPS29 495 to 745 (+)  **Database location** 1: 1253084 to 1253338 (-)  **Genomic location** 1: 1253084 to 1253338 (-)  **Alignment score** 381  **E-value** 2e-103  **Alignment length** 255  **Percentage identity** 93.73  seq_UPS29: 495 GCCCCTGCCCCGCGCCTGTGCACACACACACGTGCCACCGGGGATGGCCCCTGCCCCAAG seq_UPS29: 554  1 \|\|\|\|\| \|\|\|\|\| \|\|\|\|\|\|\|\|\|\|\|\|\|\|\|\|\|\|\|\|\|\|\|\|\|\|\|\|\|\|\|\|\|\|\|\|\|\| \|\|\|\|\| \| 60  1:1253338 GCCCCGGCCCCAAGCCTGTGCACACACACACGTGCCACCGGGGATGGCCCCCGCCCCGTG 1:1253279  seq_UPS29: 555 CCCGTGCACACA----TGTGCCACCGGGGATGGCCCCTGCCCCGCGCCTGTGCACACACA seq_UPS29: 614  61 \|\| \|\|\|\|\|\|\|\|\| \|\|\|\|\|\|\|\|\|\|\|\|\|\|\|\|\|\|\|\|\|\|\|\|\|\| \|\|\|\|\|\|\|\|\|\|\|\|\|\|\|\| 120  1:1253278 CCTGTGCACACACACACGTGCCACCGGGGATGGCCCCTGCCCCACGCCTGTGCACACACA 1:1253219  seq_UPS29: 615 CACGTGCCACTGGGGATGGCCCCTGCCCCGCGCCTGTGCACACACACACGTGCCACTGGG seq_UPS29: 674  121 \|\|\|\|\|\|\|\|\|\| \|\|\|\|\|\|\|\|\|\|\|\|\|\|\|\|\|\| \|\|\|\|\|\|\|\|\|\|\|\|\|\|\|\|\|\|\|\|\|\|\|\|\|\| \|\|\| 180  1:1253218 CACGTGCCACCGGGGATGGCCCCTGCCCCACGCCTGTGCACACACACACGTGCCACCGGG 1:1253159  seq_UPS29: 675 GATGGCCCCTGCCCCGTGCCGGTGCACACACCTACGTGCCACTGGGGATGGCCCCTGCCC seq_UPS29: 734  181 \|\|\|\|\|\|\|\|\|\|\|\|\|\|\|\|\|\|\|\|\|\|\|\|\|\|\|\|\|\|\|\|\|\|\|\|\|\|\|\|\|\|\|\|\|\|\|\|\|\|\|\|\|\|\|\|\|\|\|\| 240  1:1253158 GATGGCCCCTGCCCCGTGCCGGTGCACACACCTACGTGCCACTGGGGATGGCCCCTGCCC 1:1253099  seq_UPS29: 735 CACACACGTGCACAC seq_UPS29: 749  241 \|\|\|\|\|\|\|\|\|\|\|\|\|\|\| 255  1:1253098 CACACACGTGCACAC 1:1253080  **BLAST/BLAT type** BLASTN  **Query location** seq_UPS29 223 to 473 (+)  **Database location** 1: 1253084 to 1253338 (-)  **Genomic location** 1: 1253084 to 1253338 (-)  **Alignment score** 350  **E-value** 8e-94  **Alignment length** 255  **Percentage identity** 92.16  seq_UPS29: 223 GCCCCTGCCCCAAGCCCGTGCACACACACACGTGCCACCGGGGATGGCCCCTGCCCCGCG seq_UPS29: 282  1 \|\|\|\|\| \|\|\|\|\|\|\|\|\|\| \|\|\|\|\|\|\|\|\|\|\|\|\|\|\|\|\|\|\|\|\|\|\|\|\|\|\|\|\|\|\|\|\|\| \|\|\|\|\|\| \| 60  1:1253338 GCCCCGGCCCCAAGCCTGTGCACACACACACGTGCCACCGGGGATGGCCCCCGCCCCGTG 1:1253279  seq_UPS29: 283 CCTGTGCACACACACACGTGCCACCGGGGATGGCCCCTGCCCCAAGCCCGTGCACACAT- seq_UPS29: 342  61 \|\|\|\|\|\|\|\|\|\|\|\|\|\|\|\|\|\|\|\|\|\|\|\|\|\|\|\|\|\|\|\|\|\|\|\|\|\|\|\|\|\|\|\| \|\|\| \|\|\|\|\|\|\|\|\| 120  1:1253278 CCTGTGCACACACACACGTGCCACCGGGGATGGCCCCTGCCCCACGCCTGTGCACACACA 1:1253219  seq_UPS29: 343 ---GTGCCACCGGGGATGGCCCCTGCCCCGCGCCTGTGCACACACACACGTGCCACTGGG seq_UPS29: 402  121 \|\|\|\|\|\|\|\|\|\|\|\|\|\|\|\|\|\|\|\|\|\|\|\|\|\| \|\|\|\|\|\|\|\|\|\|\|\|\|\|\|\|\|\|\|\|\|\|\|\|\|\| \|\|\| 180  1:1253218 CACGTGCCACCGGGGATGGCCCCTGCCCCACGCCTGTGCACACACACACGTGCCACCGGG 1:1253159  seq_UPS29: 403 GATGGCCCCTGCCCCGTGCCTGTGCACACACACACGTGCCACCGGGGATGGCCCCTGCCC seq_UPS29: 462  181 \|\|\|\|\|\|\|\|\|\|\|\|\|\|\|\|\|\|\|\| \|\|\|\|\|\|\|\|\|\| \|\|\|\|\|\|\|\|\| \|\|\|\|\|\|\|\|\|\|\|\|\|\|\|\|\| 240  1:1253158 GATGGCCCCTGCCCCGTGCCGGTGCACACACCTACGTGCCACTGGGGATGGCCCCTGCCC 1:1253099  seq_UPS29: 463 CAAGCCCGTGCACAC seq_UPS29: 477  241 \|\| \| \|\|\|\|\|\|\|\|\| 255  1:1253098 CACACACGTGCACAC 1:1253080 | | | | | | | | | | | | | | |
| Ensemble, BLAST/BLAT search **Job name** Job 3: seq_UPS29 745 bp (human)  **Species Chimpanzee (*Pan troglodytes***) 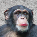  **Assembly** Pan_tro_3.0  **Search type** BLASTN (NCBI Blast) | | | | | | | | | | | | | | |
| **Genomic Location** | | **Overlapping Gene(s)** | **Orientation** | | **Query start** | | | **Query end** | | **Length** | | **Score** | **E-value** | **%ID** |
| [1:482204-482409](http://www.ensembl.org/Pan_troglodytes/Location/View?r=1:482193-482419;tl=N3LVsKKEHxXtM9se-6276219-776269167) [[Sequence]](http://www.ensembl.org/Pan_troglodytes/Tools/Blast/GenomicSeq?r=1:1297164-1297243;tl=N3LVsKKEHxXtM9se-6276219-776269167) | | [ACAP3](http://www.ensembl.org/Pan_troglodytes/Gene/Summary?db=core;g=ENSPTRG00000024081;r=1:1297164-1297243;tl=N3LVsKKEHxXtM9se-6276219-776269167) | Reverse | | 541 | | | 744 | | 206 [[Sequence]](http://www.ensembl.org/Pan_troglodytes/Tools/Blast/QuerySeq?r=1:1297164-1297243;tl=N3LVsKKEHxXtM9se-6276219-776269167) | | 336 | 1e-89 | 95.63 [[Alignment]](http://www.ensembl.org/Pan_troglodytes/Tools/Blast/Alignment?r=1:1297164-1297243;tl=N3LVsKKEHxXtM9se-6276219-776269167) |
| [1:482217-482409](http://www.ensembl.org/Pan_troglodytes/Location/View?r=1:482207-482418;tl=N3LVsKKEHxXtM9se-6276219-776269168) [[Sequence]](http://www.ensembl.org/Pan_troglodytes/Tools/Blast/GenomicSeq?r=1:1297164-1297243;tl=N3LVsKKEHxXtM9se-6276219-776269168) | | [ACAP3](http://www.ensembl.org/Pan_troglodytes/Gene/Summary?db=core;g=ENSPTRG00000024081;r=1:1297164-1297243;tl=N3LVsKKEHxXtM9se-6276219-776269168) | Reverse | | 449 | | | 639 | | 195 [[Sequence]](http://www.ensembl.org/Pan_troglodytes/Tools/Blast/QuerySeq?r=1:1297164-1297243;tl=N3LVsKKEHxXtM9se-6276219-776269168) | | 279 | 2e-72 | 92.82 [[Alignment]](http://www.ensembl.org/Pan_troglodytes/Tools/Blast/Alignment?r=1:1297164-1297243;tl=N3LVsKKEHxXtM9se-6276219-776269168) |
| [1:482217-482409](http://www.ensembl.org/Pan_troglodytes/Location/View?r=1:482207-482418;tl=N3LVsKKEHxXtM9se-6276219-776269169) [[Sequence]](http://www.ensembl.org/Pan_troglodytes/Tools/Blast/GenomicSeq?r=1:1297164-1297243;tl=N3LVsKKEHxXtM9se-6276219-776269169) | | [ACAP3](http://www.ensembl.org/Pan_troglodytes/Gene/Summary?db=core;g=ENSPTRG00000024081;r=1:1297164-1297243;tl=N3LVsKKEHxXtM9se-6276219-776269169) | Reverse | | 223 | | | 413 | | 195 [[Sequence]](http://www.ensembl.org/Pan_troglodytes/Tools/Blast/QuerySeq?r=1:1297164-1297243;tl=N3LVsKKEHxXtM9se-6276219-776269169) | | 279 | 2e-72 | 92.82 [[Alignment]](http://www.ensembl.org/Pan_troglodytes/Tools/Blast/Alignment?r=1:1297164-1297243;tl=N3LVsKKEHxXtM9se-6276219-776269169) |
| [1:482216-482389](http://www.ensembl.org/Pan_troglodytes/Location/View?r=1:482207-482397;tl=N3LVsKKEHxXtM9se-6276219-776269170) [[Sequence]](http://www.ensembl.org/Pan_troglodytes/Tools/Blast/GenomicSeq?r=1:1297164-1297243;tl=N3LVsKKEHxXtM9se-6276219-776269170) | | [ACAP3](http://www.ensembl.org/Pan_troglodytes/Gene/Summary?db=core;g=ENSPTRG00000024081;r=1:1297164-1297243;tl=N3LVsKKEHxXtM9se-6276219-776269170) | Reverse | | 379 | | | 552 | | 174 [[Sequence]](http://www.ensembl.org/Pan_troglodytes/Tools/Blast/QuerySeq?r=1:1297164-1297243;tl=N3LVsKKEHxXtM9se-6276219-776269170) | | 273 | 1e-70 | 94.83 [[Alignment]](http://www.ensembl.org/Pan_troglodytes/Tools/Blast/Alignment?r=1:1297164-1297243;tl=N3LVsKKEHxXtM9se-6276219-776269170) |
| [1:482216-482389](http://www.ensembl.org/Pan_troglodytes/Location/View?r=1:482207-482397;tl=N3LVsKKEHxXtM9se-6276219-776269171) [[Sequence]](http://www.ensembl.org/Pan_troglodytes/Tools/Blast/GenomicSeq?r=1:1297164-1297243;tl=N3LVsKKEHxXtM9se-6276219-776269171) | | [ACAP3](http://www.ensembl.org/Pan_troglodytes/Gene/Summary?db=core;g=ENSPTRG00000024081;r=1:1297164-1297243;tl=N3LVsKKEHxXtM9se-6276219-776269171) | Reverse | | 153 | | | 326 | | 174 [[Sequence]](http://www.ensembl.org/Pan_troglodytes/Tools/Blast/QuerySeq?r=1:1297164-1297243;tl=N3LVsKKEHxXtM9se-6276219-776269171) | | 273 | 1e-70 | 94.83 [[Alignment]](http://www.ensembl.org/Pan_troglodytes/Tools/Blast/Alignment?r=1:1297164-1297243;tl=N3LVsKKEHxXtM9se-6276219-776269171) |
| [1:482217-482409](http://www.ensembl.org/Pan_troglodytes/Location/View?r=1:482207-482418;tl=N3LVsKKEHxXtM9se-6276219-776269172) [[Sequence]](http://www.ensembl.org/Pan_troglodytes/Tools/Blast/GenomicSeq?r=1:1297164-1297243;tl=N3LVsKKEHxXtM9se-6276219-776269172) | | [ACAP3](http://www.ensembl.org/Pan_troglodytes/Gene/Summary?db=core;g=ENSPTRG00000024081;r=1:1297164-1297243;tl=N3LVsKKEHxXtM9se-6276219-776269172) | Reverse | | 1 | | | 187 | | 193 [[Sequence]](http://www.ensembl.org/Pan_troglodytes/Tools/Blast/QuerySeq?r=1:1297164-1297243;tl=N3LVsKKEHxXtM9se-6276219-776269172) | | 271 | 5e-70 | 92.23 [[Alignment]](http://www.ensembl.org/Pan_troglodytes/Tools/Blast/Alignment?r=1:1297164-1297243;tl=N3LVsKKEHxXtM9se-6276219-776269172) |
| [1:482217-482409](http://www.ensembl.org/Pan_troglodytes/Location/View?r=1:482207-482418;tl=N3LVsKKEHxXtM9se-6276219-776269173) [[Sequence]](http://www.ensembl.org/Pan_troglodytes/Tools/Blast/GenomicSeq?r=1:1297164-1297243;tl=N3LVsKKEHxXtM9se-6276219-776269173) | | [ACAP3](http://www.ensembl.org/Pan_troglodytes/Gene/Summary?db=core;g=ENSPTRG00000024081;r=1:1297164-1297243;tl=N3LVsKKEHxXtM9se-6276219-776269173) | Reverse | | 315 | | | 505 | | 193 [[Sequence]](http://www.ensembl.org/Pan_troglodytes/Tools/Blast/QuerySeq?r=1:1297164-1297243;tl=N3LVsKKEHxXtM9se-6276219-776269173) | | 263 | 1e-67 | 92.23 [[Alignment]](http://www.ensembl.org/Pan_troglodytes/Tools/Blast/Alignment?r=1:1297164-1297243;tl=N3LVsKKEHxXtM9se-6276219-776269173) |
| [1:482217-482385](http://www.ensembl.org/Pan_troglodytes/Location/View?r=1:482208-482393;tl=N3LVsKKEHxXtM9se-6276219-776269174) [[Sequence]](http://www.ensembl.org/Pan_troglodytes/Tools/Blast/GenomicSeq?r=1:1297164-1297243;tl=N3LVsKKEHxXtM9se-6276219-776269174) | | [ACAP3](http://www.ensembl.org/Pan_troglodytes/Gene/Summary?db=core;g=ENSPTRG00000024081;r=1:1297164-1297243;tl=N3LVsKKEHxXtM9se-6276219-776269174) | Reverse | | 111 | | | 279 | | 169 [[Sequence]](http://www.ensembl.org/Pan_troglodytes/Tools/Blast/QuerySeq?r=1:1297164-1297243;tl=N3LVsKKEHxXtM9se-6276219-776269174) | | 247 | 7e-63 | 93.49 [[Alignment]](http://www.ensembl.org/Pan_troglodytes/Tools/Blast/Alignment?r=1:1297164-1297243;tl=N3LVsKKEHxXtM9se-6276219-776269174) |
| [1:482216-482389](http://www.ensembl.org/Pan_troglodytes/Location/View?r=1:482207-482397;tl=N3LVsKKEHxXtM9se-6276219-776269175) [[Sequence]](http://www.ensembl.org/Pan_troglodytes/Tools/Blast/GenomicSeq?r=1:1297164-1297243;tl=N3LVsKKEHxXtM9se-6276219-776269175) | | [ACAP3](http://www.ensembl.org/Pan_troglodytes/Gene/Summary?db=core;g=ENSPTRG00000024081;r=1:1297164-1297243;tl=N3LVsKKEHxXtM9se-6276219-776269175) | Reverse | | 67 | | | 234 | | 174 [[Sequence]](http://www.ensembl.org/Pan_troglodytes/Tools/Blast/QuerySeq?r=1:1297164-1297243;tl=N3LVsKKEHxXtM9se-6276219-776269175) | | 241 | 4e-61 | 91.95 [[Alignment]](http://www.ensembl.org/Pan_troglodytes/Tools/Blast/Alignment?r=1:1297164-1297243;tl=N3LVsKKEHxXtM9se-6276219-776269175) |
| [1:482217-482389](http://www.ensembl.org/Pan_troglodytes/Location/View?r=1:482208-482397;tl=N3LVsKKEHxXtM9se-6276219-776269176) [[Sequence]](http://www.ensembl.org/Pan_troglodytes/Tools/Blast/GenomicSeq?r=1:1297164-1297243;tl=N3LVsKKEHxXtM9se-6276219-776269176) | | [ACAP3](http://www.ensembl.org/Pan_troglodytes/Gene/Summary?db=core;g=ENSPTRG00000024081;r=1:1297164-1297243;tl=N3LVsKKEHxXtM9se-6276219-776269176) | Reverse | | 425 | | | 593 | | 173 [[Sequence]](http://www.ensembl.org/Pan_troglodytes/Tools/Blast/QuerySeq?r=1:1297164-1297243;tl=N3LVsKKEHxXtM9se-6276219-776269176) | | 239 | 2e-60 | 92.49 [[Alignment]](http://www.ensembl.org/Pan_troglodytes/Tools/Blast/Alignment?r=1:1297164-1297243;tl=N3LVsKKEHxXtM9se-6276219-776269176) |
| [1:482217-482389](http://www.ensembl.org/Pan_troglodytes/Location/View?r=1:482208-482397;tl=N3LVsKKEHxXtM9se-6276219-776269177) [[Sequence]](http://www.ensembl.org/Pan_troglodytes/Tools/Blast/GenomicSeq?r=1:1297164-1297243;tl=N3LVsKKEHxXtM9se-6276219-776269177) | | [ACAP3](http://www.ensembl.org/Pan_troglodytes/Gene/Summary?db=core;g=ENSPTRG00000024081;r=1:1297164-1297243;tl=N3LVsKKEHxXtM9se-6276219-776269177) | Reverse | | 199 | | | 367 | | 173 [[Sequence]](http://www.ensembl.org/Pan_troglodytes/Tools/Blast/QuerySeq?r=1:1297164-1297243;tl=N3LVsKKEHxXtM9se-6276219-776269177) | | 239 | 2e-60 | 92.49 [[Alignment]](http://www.ensembl.org/Pan_troglodytes/Tools/Blast/Alignment?r=1:1297164-1297243;tl=N3LVsKKEHxXtM9se-6276219-776269177) |
| [1:482216-482389](http://www.ensembl.org/Pan_troglodytes/Location/View?r=1:482207-482397;tl=N3LVsKKEHxXtM9se-6276219-776269178) [[Sequence]](http://www.ensembl.org/Pan_troglodytes/Tools/Blast/GenomicSeq?r=1:1297164-1297243;tl=N3LVsKKEHxXtM9se-6276219-776269178) | | [ACAP3](http://www.ensembl.org/Pan_troglodytes/Gene/Summary?db=core;g=ENSPTRG00000024081;r=1:1297164-1297243;tl=N3LVsKKEHxXtM9se-6276219-776269178) | Reverse | | 291 | | | 460 | | 174 [[Sequence]](http://www.ensembl.org/Pan_troglodytes/Tools/Blast/QuerySeq?r=1:1297164-1297243;tl=N3LVsKKEHxXtM9se-6276219-776269178) | | 229 | 2e-57 | 91.38 [[Alignment]](http://www.ensembl.org/Pan_troglodytes/Tools/Blast/Alignment?r=1:1297164-1297243;tl=N3LVsKKEHxXtM9se-6276219-776269178) |
| [1:482217-482389](http://www.ensembl.org/Pan_troglodytes/Location/View?r=1:482208-482397;tl=N3LVsKKEHxXtM9se-6276219-776269179) [[Sequence]](http://www.ensembl.org/Pan_troglodytes/Tools/Blast/GenomicSeq?r=1:1297164-1297243;tl=N3LVsKKEHxXtM9se-6276219-776269179) | | [ACAP3](http://www.ensembl.org/Pan_troglodytes/Gene/Summary?db=core;g=ENSPTRG00000024081;r=1:1297164-1297243;tl=N3LVsKKEHxXtM9se-6276219-776269179) | Reverse | | 517 | | | 685 | | 173 [[Sequence]](http://www.ensembl.org/Pan_troglodytes/Tools/Blast/QuerySeq?r=1:1297164-1297243;tl=N3LVsKKEHxXtM9se-6276219-776269179) | | 227 | 6e-57 | 91.33 [[Alignment]](http://www.ensembl.org/Pan_troglodytes/Tools/Blast/Alignment?r=1:1297164-1297243;tl=N3LVsKKEHxXtM9se-6276219-776269179) |
| [1:482217-482365](http://www.ensembl.org/Pan_troglodytes/Location/View?r=1:482209-482372;tl=N3LVsKKEHxXtM9se-6276219-776269180) [[Sequence]](http://www.ensembl.org/Pan_troglodytes/Tools/Blast/GenomicSeq?r=1:1297164-1297243;tl=N3LVsKKEHxXtM9se-6276219-776269180) | | [ACAP3](http://www.ensembl.org/Pan_troglodytes/Gene/Summary?db=core;g=ENSPTRG00000024081;r=1:1297164-1297243;tl=N3LVsKKEHxXtM9se-6276219-776269180) | Reverse | | 1 | | | 141 | | 149 [[Sequence]](http://www.ensembl.org/Pan_troglodytes/Tools/Blast/QuerySeq?r=1:1297164-1297243;tl=N3LVsKKEHxXtM9se-6276219-776269180) | | 184 | 8e-44 | 89.93 [[Alignment]](http://www.ensembl.org/Pan_troglodytes/Tools/Blast/Alignment?r=1:1297164-1297243;tl=N3LVsKKEHxXtM9se-6276219-776269180) |
| [1:482263-482389](http://www.ensembl.org/Pan_troglodytes/Location/View?r=1:482256-482395;tl=N3LVsKKEHxXtM9se-6276219-776269181) [[Sequence]](http://www.ensembl.org/Pan_troglodytes/Tools/Blast/GenomicSeq?r=1:1297164-1297243;tl=N3LVsKKEHxXtM9se-6276219-776269181) | | [ACAP3](http://www.ensembl.org/Pan_troglodytes/Gene/Summary?db=core;g=ENSPTRG00000024081;r=1:1297164-1297243;tl=N3LVsKKEHxXtM9se-6276219-776269181) | Reverse | | 605 | | | 731 | | 127 [[Sequence]](http://www.ensembl.org/Pan_troglodytes/Tools/Blast/QuerySeq?r=1:1297164-1297243;tl=N3LVsKKEHxXtM9se-6276219-776269181) | | 172 | 3e-40 | 92.13 [[Alignment]](http://www.ensembl.org/Pan_troglodytes/Tools/Blast/Alignment?r=1:1297164-1297243;tl=N3LVsKKEHxXtM9se-6276219-776269181) |
| [1:482217-482319](http://www.ensembl.org/Pan_troglodytes/Location/View?r=1:482211-482324;tl=N3LVsKKEHxXtM9se-6276219-776269182) [[Sequence]](http://www.ensembl.org/Pan_troglodytes/Tools/Blast/GenomicSeq?r=1:1297164-1297243;tl=N3LVsKKEHxXtM9se-6276219-776269182) | | [ACAP3](http://www.ensembl.org/Pan_troglodytes/Gene/Summary?db=core;g=ENSPTRG00000024081;r=1:1297164-1297243;tl=N3LVsKKEHxXtM9se-6276219-776269182) | Reverse | | 1 | | | 101 | | 103 [[Sequence]](http://www.ensembl.org/Pan_troglodytes/Tools/Blast/QuerySeq?r=1:1297164-1297243;tl=N3LVsKKEHxXtM9se-6276219-776269182) | | 132 | 2e-28 | 91.26 [[Alignment]](http://www.ensembl.org/Pan_troglodytes/Tools/Blast/Alignment?r=1:1297164-1297243;tl=N3LVsKKEHxXtM9se-6276219-776269182) |
| [1:482307-482389](http://www.ensembl.org/Pan_troglodytes/Location/View?r=1:482302-482393;tl=N3LVsKKEHxXtM9se-6276219-776269183) [[Sequence]](http://www.ensembl.org/Pan_troglodytes/Tools/Blast/GenomicSeq?r=1:1297164-1297243;tl=N3LVsKKEHxXtM9se-6276219-776269183) | | [ACAP3](http://www.ensembl.org/Pan_troglodytes/Gene/Summary?db=core;g=ENSPTRG00000024081;r=1:1297164-1297243;tl=N3LVsKKEHxXtM9se-6276219-776269183) | Reverse | | 651 | | | 733 | | 83 [[Sequence]](http://www.ensembl.org/Pan_troglodytes/Tools/Blast/QuerySeq?r=1:1297164-1297243;tl=N3LVsKKEHxXtM9se-6276219-776269183) | | 125 | 5e-26 | 93.98 [[Alignment]](http://www.ensembl.org/Pan_troglodytes/Tools/Blast/Alignment?r=1:1297164-1297243;tl=N3LVsKKEHxXtM9se-6276219-776269183) |
| [1:482217-482273](http://www.ensembl.org/Pan_troglodytes/Location/View?r=1:482214-482275;tl=N3LVsKKEHxXtM9se-6276219-776269184) [[Sequence]](http://www.ensembl.org/Pan_troglodytes/Tools/Blast/GenomicSeq?r=1:1297164-1297243;tl=N3LVsKKEHxXtM9se-6276219-776269184) | | [ACAP3](http://www.ensembl.org/Pan_troglodytes/Gene/Summary?db=core;g=ENSPTRG00000024081;r=1:1297164-1297243;tl=N3LVsKKEHxXtM9se-6276219-776269184) | Reverse | | 1 | | | 55 | | 57 [[Sequence]](http://www.ensembl.org/Pan_troglodytes/Tools/Blast/QuerySeq?r=1:1297164-1297243;tl=N3LVsKKEHxXtM9se-6276219-776269184) | | 65.7 | 4e-08 | 89.47 [[Alignment]](http://www.ensembl.org/Pan_troglodytes/Tools/Blast/Alignment?r=1:1297164-1297243;tl=N3LVsKKEHxXtM9se-6276219-776269184) |
| [1:482355-482409](http://www.ensembl.org/Pan_troglodytes/Location/View?r=1:482352-482411;tl=N3LVsKKEHxXtM9se-6276219-776269185) [[Sequence]](http://www.ensembl.org/Pan_troglodytes/Tools/Blast/GenomicSeq?r=1:1297164-1297243;tl=N3LVsKKEHxXtM9se-6276219-776269185) | | [ACAP3](http://www.ensembl.org/Pan_troglodytes/Gene/Summary?db=core;g=ENSPTRG00000024081;r=1:1297164-1297243;tl=N3LVsKKEHxXtM9se-6276219-776269185) | Reverse | | 675 | | | 731 | | 57 [[Sequence]](http://www.ensembl.org/Pan_troglodytes/Tools/Blast/QuerySeq?r=1:1297164-1297243;tl=N3LVsKKEHxXtM9se-6276219-776269185) | | 57.8 | 9e-06 | 87.72 [[Alignment]](http://www.ensembl.org/Pan_troglodytes/Tools/Blast/Alignment?r=1:1297164-1297243;tl=N3LVsKKEHxXtM9se-6276219-776269185) |
| BLAST/BLAT Alignment **BLAST/BLAT type** BLASTN  **Query location** seq_UPS29 541 to 744 (+)  **Database location** 1: 482204 to 482409 (-)  **Genomic location** 1: 482204 to 482409 (-)  **Alignment score** 336  **E-value** 1e-89  **Alignment length** 206  **Percentage identity** 95.63  seq_UPS29: 541 GCCCCTGCCCCAAGCCCGTGCACACA--TGTGCCACCGGGGATGGCCCCTGCCCCGCGCC seq_UPS29: 600  1 \|\|\|\|\| \|\|\|\|\|\|\|\|\|\|\|\|\|\|\|\|\|\|\|\| \|\|\|\|\|\|\|\|\|\|\|\|\|\|\|\|\|\|\|\|\|\|\|\|\|\|\| \|\|\| 60  1:482409 GCCCCGGCCCCAAGCCCGTGCACACACACGTGCCACCGGGGATGGCCCCTGCCCCGTGCC 1:482350  seq_UPS29: 601 TGTGCACACACACACGTGCCACTGGGGATGGCCCCTGCCCCGCGCCTGTGCACACACACA seq_UPS29: 660  61 \|\|\|\|\|\|\|\|\|\|\|\|\|\|\|\|\|\|\|\|\|\| \|\|\|\|\|\|\|\|\|\|\|\|\|\|\|\|\|\| \|\|\|\|\|\|\|\|\|\|\|\|\|\|\|\|\|\| 120  1:482349 TGTGCACACACACACGTGCCACCGGGGATGGCCCCTGCCCCACGCCTGTGCACACACACA 1:482290  seq_UPS29: 661 CGTGCCACTGGGGATGGCCCCTGCCCCGTGCCGGTGCACACACCTACGTGCCACTGGGGA seq_UPS29: 720  121 \|\|\|\|\|\|\| \|\|\|\|\|\|\|\|\|\|\|\|\|\|\|\|\|\|\|\|\|\|\|\|\|\|\|\|\|\|\|\|\|\|\|\|\|\|\|\|\|\|\|\|\|\|\|\|\|\|\| 180  1:482289 TGTGCCACCGGGGATGGCCCCTGCCCCGTGCCGGTGCACACACCTACGTGCCACTGGGGA 1:482230  seq_UPS29: 721 TGGCCCCTGCCCCACACACGTGCACA seq_UPS29: 746  181 \|\|\|\|\|\|\|\|\|\|\|\|\|\|\|\|\|\|\|\|\|\|\|\|\|\| 206  1:482229 TGGCCCCTGCCCCACACACGTGCACA 1:482202  **BLAST/BLAT type** BLASTN  **Query location** seq_UPS29 449 to 639 (+)  **Database location** 1: 482217 to 482409 (-)  **Genomic location** 1: 482217 to 482409 (-)  **Alignment score** 279  **E-value** 2e-72  **Alignment length** 195  **Percentage identity** 92.82  seq_UPS29: 449 GCCCCTGCCCCAAGCCCGTGCACACACACACGTGCCACCGGGGATGGCCCCTGCCCCGCG seq_UPS29: 508  1 \|\|\|\|\| \|\|\|\|\|\|\|\|\|\|\|\|\|\|\|\|\|\|\|\|\|\|\| \|\|\|\|\|\|\|\|\|\|\|\|\|\|\|\|\|\|\|\|\|\|\|\|\|\|\| \| 60  1:482409 GCCCCGGCCCCAAGCCCGTGCACACACAC--GTGCCACCGGGGATGGCCCCTGCCCCGTG 1:482350  seq_UPS29: 509 CCTGTGCACACACACACGTGCCACCGGGGATGGCCCCTGCCCCAAGCCCGTGCACACA-- seq_UPS29: 568  61 \|\|\|\|\|\|\|\|\|\|\|\|\|\|\|\|\|\|\|\|\|\|\|\|\|\|\|\|\|\|\|\|\|\|\|\|\|\|\|\|\|\|\|\| \|\|\| \|\|\|\|\|\|\|\|\| 120  1:482349 CCTGTGCACACACACACGTGCCACCGGGGATGGCCCCTGCCCCACGCCTGTGCACACACA 1:482290  seq_UPS29: 569 --TGTGCCACCGGGGATGGCCCCTGCCCCGCGCCTGTGCACACACACACGTGCCACTGGG seq_UPS29: 628  121 \|\|\|\|\|\|\|\|\|\|\|\|\|\|\|\|\|\|\|\|\|\|\|\|\|\|\|\| \|\|\| \|\|\|\|\|\|\|\|\|\| \|\|\|\|\|\|\|\|\|\|\|\|\| 180  1:482289 CATGTGCCACCGGGGATGGCCCCTGCCCCGTGCCGGTGCACACACCTACGTGCCACTGGG 1:482230  seq_UPS29: 629 GATGGCCCCTGCCCC seq_UPS29: 643  181 \|\|\|\|\|\|\|\|\|\|\|\|\|\|\| 195  1:482229 GATGGCCCCTGCCCC 1:482213  **BLAST/BLAT type** BLASTN  **Query location** seq_UPS29 223 to 413 (+)  **Database location** 1: 482217 to 482409 (-)  **Genomic location** 1: 482217 to 482409 (-)  **Alignment score** 279  **E-value** 2e-72  **Alignment length** 195  **Percentage identity** 92.82  seq_UPS29: 223 GCCCCTGCCCCAAGCCCGTGCACACACACACGTGCCACCGGGGATGGCCCCTGCCCCGCG seq_UPS29: 282  1 \|\|\|\|\| \|\|\|\|\|\|\|\|\|\|\|\|\|\|\|\|\|\|\|\|\|\|\| \|\|\|\|\|\|\|\|\|\|\|\|\|\|\|\|\|\|\|\|\|\|\|\|\|\|\| \| 60  1:482409 GCCCCGGCCCCAAGCCCGTGCACACACAC--GTGCCACCGGGGATGGCCCCTGCCCCGTG 1:482350  seq_UPS29: 283 CCTGTGCACACACACACGTGCCACCGGGGATGGCCCCTGCCCCAAGCCCGTGCACACA-- seq_UPS29: 342  61 \|\|\|\|\|\|\|\|\|\|\|\|\|\|\|\|\|\|\|\|\|\|\|\|\|\|\|\|\|\|\|\|\|\|\|\|\|\|\|\|\|\|\|\| \|\|\| \|\|\|\|\|\|\|\|\| 120  1:482349 CCTGTGCACACACACACGTGCCACCGGGGATGGCCCCTGCCCCACGCCTGTGCACACACA 1:482290  seq_UPS29: 343 --TGTGCCACCGGGGATGGCCCCTGCCCCGCGCCTGTGCACACACACACGTGCCACTGGG seq_UPS29: 402  121 \|\|\|\|\|\|\|\|\|\|\|\|\|\|\|\|\|\|\|\|\|\|\|\|\|\|\|\| \|\|\| \|\|\|\|\|\|\|\|\|\| \|\|\|\|\|\|\|\|\|\|\|\|\| 180  1:482289 CATGTGCCACCGGGGATGGCCCCTGCCCCGTGCCGGTGCACACACCTACGTGCCACTGGG 1:482230  seq_UPS29: 403 GATGGCCCCTGCCCC seq_UPS29: 417  181 \|\|\|\|\|\|\|\|\|\|\|\|\|\|\| 195  1:482229 GATGGCCCCTGCCCC 1:482213 | | | | | | | | | | | | | | |


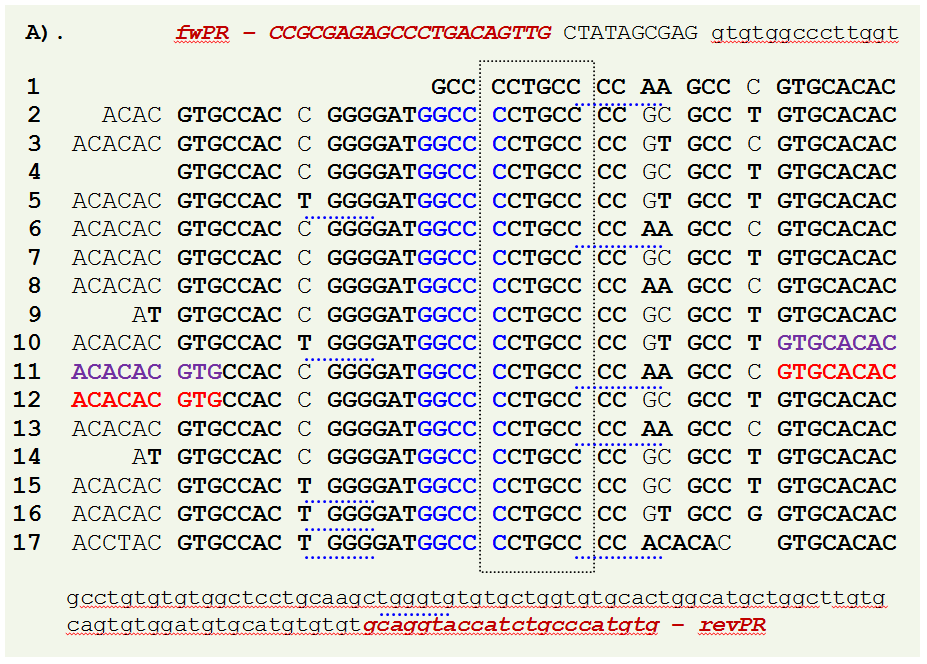


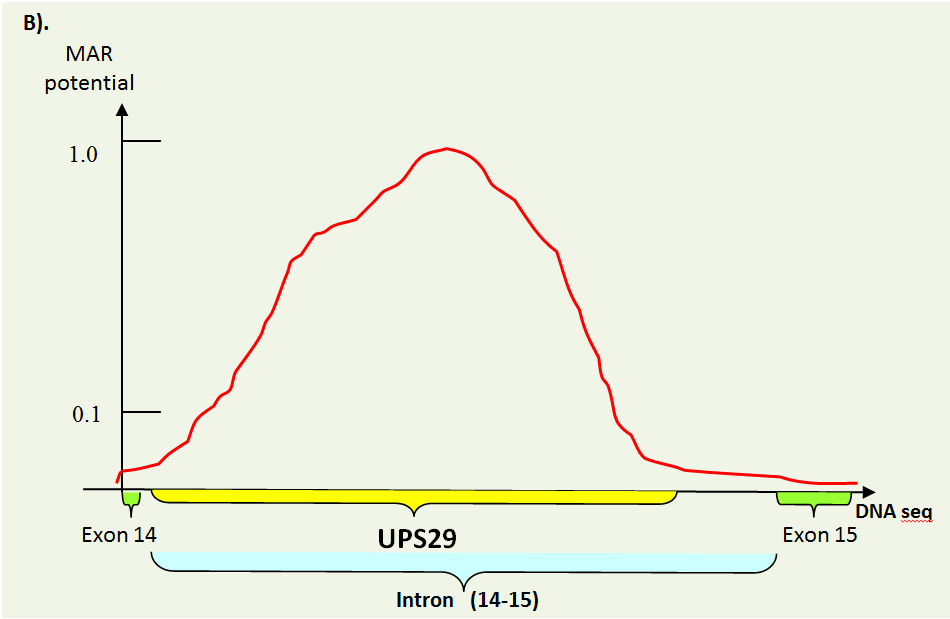


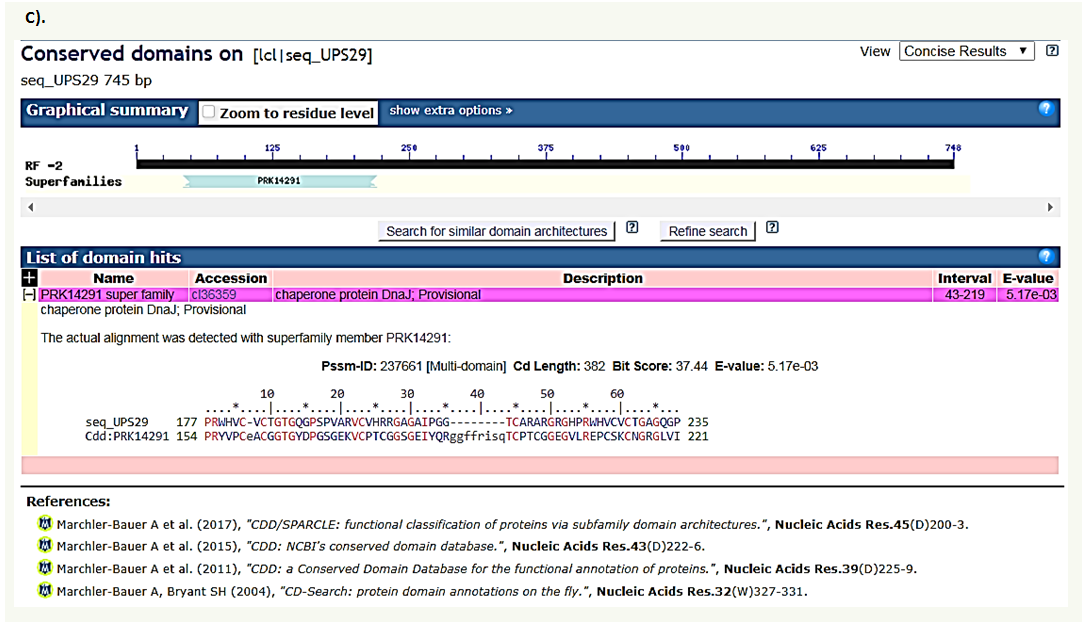


Figure S1. *In silico* analysis of UPS29 (search of potential sites of DNA instability and conserved domains). (A) Fragment of the nucleotide sequence of the human *ACAP3* gene containing UPS29 (allele of 17 repeats). Lowercase letters indicate the nucleotide sequence of the intron in which UPS29 is located. “1-17” - UPS29 repeats. Sites contributed to the of human minisatellites instability in the germ line [Boan et al., 1997] are marked in the dashed rectangle. Sites homologous to one of the recombination signals for immunoglobulin class switching [Weinreb et al., 1988] are underlined by a blue dashed line. Potential DNA curvature sites are marked with blue letters; area with MAR potential = 1.0 is marked in red letters; area with MAR potential = 0.8-0.9 is marked in purple letters [Mar-Wiz, accessed 2003]. Areas of the primers (fwPR and revPR) annealing are indicated in brown letters. (B) MAR potential of nucleotide sequences consisting of exon 14, intron 14-15 and exon 15 of *ACAP3* gene [Mar-Wiz, accessed 2003]. Potential sites of kinked DNA into UPS29 are in every repeats (5’-GTGCACACACACGTG-3’). (C) Results of search for conserved domains into UPS29 sequence (NCBI Conserved Domain Search, https://www.ncbi.nlm.nih.gov/Structure/cdd/wrpsb.cgi, accessed 07.06.2020). It was revealed homology with chaperone protein DnaJ (PRK14291 super family, accession [cl36359](https://www.ncbi.nlm.nih.gov/Structure/cdd/cddsrv.cgi?ascbin=8&maxaln=10&seltype=2&uid=cl36359)) (area between 4 and 6 repeats of UPS29).

Table S3. Sequencing results of PCR products of human UPS29 minisatellite.

| Sequencing results of UPS29 PCR products  from seven individuals without epilepsy |
| --- |
| **Long allele (17 repeats), ~900 bp PCR-band in 6% PAAG:**  *primer* *fwPR*: del- **TATAG**C GAG del-gtgtggcccttggt  **1/2** AGGC **G**N**GCCAC** C **GGGGA--G CCTCCGCCCC** **AA GCC** C **GTGC ACAC = a/b**  **2** ACAC **G**N**GCCAC** C **GGGGATGG CCCCTGCCCC** GC **GCC T GTGC ACAC = b**  **3** ACACAC **GTGCCAC** C **GGGGATGG CCCCTGCCCC** G**T GCC** C **GTGC ACAC = c**  **4 GTGCCAC** C **GGGGATGG CCCCTGCCCC** GC **GCC T GTGC ACAC = d** 5 ACACAC GTGCCAC T GGGGATGG CCCCTGCCCC GT GCC T GTGC ACAC = e6 ACACAC GTGCCAC C GGGGATGG CCCCTGCCCC AA GCC C GTGC ACAC = f7 ACACAC GTGCCAC C GGGGATGG CCCCTGCCCC GC GCC T GTGCCACAC = g8 ACACAC GTGCCAC C GGGGATGG CCCCTGCCCC AA GCC C TGCG………… = f **9 ……………………………………………………………………………………………………………………………………………… = h**  **10 ……………………………………………………………………………………………………………………………………………… = e**  **11** ACACAC **GTGCCAC** C GG**GGATGG CCCCTGCCCC**C**AA GCC** C **GTGCACAC = f**  **12** ACACAC **GTGCCAC** C GG**GGATGG CCCCTGCCCC** GC **GCC T GTGCACAC = g**  **13** ACACAC **GTGCCAC** C GG**GGATGG CCCCTGCCCC AA GCC** C **GTGCACAC = f**  **14** AT **GTGCCAC** C GG**GGATGG CCCCTGCCCC** GC **GCC T GTGCACAC = h**  **15** ACACAC **GTGCCAC T GGGGATGG CCCCTGCCCC** GC **GCC T GTGCACAC = i**  **16** ACACAC **GTGCCAC T GGGGATGG CCCCTGCCCC** G**T G**CC G G**TGCACAC = j**  **17** AC**CT**AC **GTGCCAC T GGGGATGG CCCCTGCCCC** ACACAC **GTGCACAC = k**  gcctgtgtgtggctcctgcaagctgggtgtgtgctggtgtgcactggcaNgctg  del-gcttgtgcagtgtggatgtgcatgtgtgt : *primer revRP*  Repeated units 9 and 10 are absent because of breaking up the sequencing.  **Long allele (17 repeats), ~900 bp PCR-band in 6% PAAG:**  *primer* *fwPR*: del- **TATAG** CGAG gtgtggcccttggt  **1 GCCCCTGCCCC AA GCC** C **GTGCACAC = a**  **2** ACAC **GTG CCAC** C **GGGGATGGCCCCTGCCCC** GC **GCC T GTGCACAC = b**  **3** ACACAC **GTG CCAC** C **GGGGATGGCCCCTGCCCC** G**T GCC** C **GTGCACAC = c**  **4 GTGTCCAC** C **GGGGATGGCCCCTGCCCC** GC **GCC T GTGCACAC = d** (ins **T**)  **5** ACACAC **GTG CCAC T GGGGATGGCCCCTGCCCC** G**T GCC T GTGCACAC = e**  **6** ACACAC **GTG CCAC** C **GGGGATGGCCCCTGCCCC AA GCC** C **GTGCACAC = f**  **7** ACACAC **GTG CCAC** C **GGGGATGGCCCCTGCCCC** GC **GCC T GTGCACAC = g**  **8** ACACAC **GTG CCAC** C **GGGGATGGCCCCTGCCCC AA GCC** C **GTGCACAC = f**  **9** AT **GTG CCAC** C **GGGGATGGCCCCTGCCCC** GC **GCC T GTGCACAC = h**  **10** ACACAC **GTG CCAC T GGGGATGGCCCCTGCCCC** G**T GCC T GTGCACAC = e**  **11** ACACAC **GTG CCAC – GGGGATGGCCCCTGCCCC AA GCC** C **GTGCACAC = f** (1-nt del)  **12** ACACAC **GTG CCAC – GGGGATGGCC …………………………………………………………………** = g? (1-nt del)  **13 ………………………………………………………………………………………………………………………………………………**  **14 ……………………………………………………………………** **CCCTGCCCC** GC **GCC T GTGCACAC = h**  **15** ACACAC **GTG CCAC T GGGGATGGCCCCTGCCCC** GC **GCC T GTGCACAC = i**  **16** ACACAC **GTG CCAC T GGGGATGGCCCCTGCCCC** G**T GCC G GTGCACAC = j**  **17** AC**CT**AC **GTG CCAC T GGGGATGGCCCCTGCCCC** ACACAC **GTGCACAC** = **k**  gcctgtgtgtggctcctgcaagctgggtgtgtgctggtgtgcactggcatgctgcttgtgcagtgtgg  del- atgtgcatgtgtgt**:** *primer revRP*  Repeated unit 13, part of repeats 12 and 14 are absent because of breaking up the sequencing.  **Short allele (9 repeats), ~520 bp PCR-band in 6% PAAG:**  *primer* *fwPR****:* del- TATAGCGAG gtg** tggcccttggt  **1 GCCCCTGCCCC AA GTCC** C **GTGCACAC = a**(ins **Т**)  ACAC **GTGCCAC** C **GGGGATG GCCCCTGCCCC AA G CC** C **GTGCACAC = b+f**  ACAC **GTGCCAC** C **GGGGATG GCCCCTGCCCC AA G CC** C **GTGCACAC = b+f**  ACAC **GTGCCAC** C **GGGGATG GCCCCTGCCCC** G**T G CC T GTGCACAC = b+e**  **8/13** ACACAC **GTGCCAC** C **GGGGATG GCCCCTGCCCC AA G CC – GTGCACAC = f**(del **С**)  **9/14** AT **GTGC-AG** C **GGGGATG GCCCCTGCCC- G- G CC T GTGCACAC = h**(3 del and 1 substitute)  ACAC ……………………………………………………………… **CCCC** GC **G CC** **T** **GTGCACAC** = **i or h, or g**  **16** ACACAC **GTGCCAC** **T GGGGATG GCCCCTGCCCC** G**T** **G CC G GTGCACAC** = **j**  **17** AC**CT**AC **GTGCCAC** **T** **GGGGATG GCCCCTGCCCC** ACACAC **GTGCACAC** = **k**  gcctgtgtgtggctcctgcaagctgggtgtgtgctggtgtgcactggcatgctggcttgtgcagtgtgg**t**tgt del- **gcatgtgtgt** *primer revRP* (insertion t)  **Short allele (9 repeats)**, **~510 bp PCR-band in 6% PAAG:**  *primer* *fwPR****:*** del- **TATAGCGAG** gtg tggcccttggt  **1 GCCCCTGCCCC AA GCC** C **GTGCACAC = a**  ACAC **GTGCCAC** C **GGGG ATGGCCCCTGCCCC** G**T GCC T GTGCACAC = b+e** or **E** del-AC  ACAC **GTGCCAC** C **GGGGGATGGCCCCTGCCCC AA GCC** C **GTGCACAC = b+f** or **F** del-AC, ins G  **9/14** AT **GTGCCAC** C **GGGG ATGGCCCCTGCCCC** GC **GCC T GTGCACAC = h**  ACACAC **GTGCCAC** C **GGGG ATGGCCCCTGCCCC AA GCC** C **GTGCACAC = f**  AT **GTGCCAC** C **GGGG ATGGCCCCTGCCCC** G**T GCC T GTGCACAC = (h+e)**  ACACAC……………………………………………………………………………………………………… **TGCACAC =**  **16** ACACAC **GTGCCAC T --GGATGGCCCCTGCCCC** G**T GCC G GTGCACAC** = **j** (2-nt del)  **17** AC**AC**AC **GTGCCAC** **T** **GGGGATGGCCCCTGCCCC** ACACAC **GTGCACAC = k** (CT > AC) gcctgtgtgtggctcctgcaagctgggtgtgtgctggtgtgcactggcatgctggcttgtgcagtgtgg  del- atgtgcatgtgtgt: *primer revRP*  **Short allele (8 repeats), ~ 500 bp PCR-band in 6% PAAG:**  *primer fwPR:* del TATAGC GAG gtgtggcccttggt  **1 GCCCCTGCCCC AA GCC** C **GTGCACAC = a**  ACAC **GTGCCAC** C **GGGGATGGCCCCTGCCCC** G**T GCC T GTGCACAC = b+e**  ACAC **GTGCCAC** C **GGGGATGGCCCCTGCCCC AA GCC** C **GTGCACAC = b+f**  **9/14** AT **GTGCCAC** C **GGGGATGGCCCCTGCCCC** GC **GCC T GTGCACAC = h**  ACACAC **GTGCCAC** C **GGGGATGGCCCCTGCCCC AA GCC** C **GTGCACAC = f**  AT **GTGCCAC** C **GGGGATGGCCCCTGCCCC** G**T GCC T GTGCACAC = h+e**  **16** ACACAC **GTGCCAC T GGGGATGGCCCCTGCCCC** G**T GCC G GTGCACAC = j**  **17** ACACAC **GTGCCAC T GGGGATGGCCCCTGCCCC** ACACAC **GTGCACAC = k** (CT > AC)  gcctgtgtgtggctcctgcaagactgggtgtgtg**a**ctggtgtgca**g**ctggcatgctg**t**gc**c**ttgtgcagtgtgg  del- atgtgcatgtgtgt : *primer revRP* (**a,a,g,t,c** – five 1-nt insertions)  **Short allele (6 repeats), ~ 400 bp PCR-band in 6% PAAG:**  *primer fwPR*: **del-TATAG** CGAG gtgtggcccttggt  **1** **GCCCCTGCCCC AA** **GCC** C **GTGCACAC** = **a**  **2** ACAC **GTGCCAC** C **GGGGATGGCCCCTGCCCC** GC **GCC T GTGCACAC** = **b**  **13** ACACAC **GTGCCAC** C **GGGGATGGCCCCTGCCCC AA GCC** C **GTGCACAC = f**  **14** AT **GTGCCAC C GGGGATGGCCCCTGCCCC** GC **GCC T GTGCACAC = h**  **16** ACACAC **GTGCCAC T GGGGATGGCCCCTGCCCC** G**T GCC** G **GTGCACAC = j**  **17** AC**CT**AC **GTGCCAC T GGGGATGGCCCCTGCCCC** ACACAC **GTGCACAC = k**  gcctgtgtgtggctcctgcaagctgggtgtgtgctggtgtgcactggcatgctggcttgtgcagtgtgg  del- atgtgcatgtgtgt : *primer revRP*  **Short allele (6 repeats), ~ 400 bp PCR-band in 6% PAAG:**  *primer fwPR*: **5’ del- TATAG** CGAG gtgtggcccttggt  **1 GCCCCTGCCCC AA GCC** C **GTGCACAC = a**  **2** ACAC **GTGCCA**C C GG**GGATGGCCCCTGCCCC** GC **GCC T GTGCACAC = b**  **13** ACACAC **GTGCCA**C C GG**GGATGGCCCCTGCCCC AA GCC** C **GTGCACAC = f**  **14** AT **GTGCCA**C C GG**GGATGGCCCCTGCCCC** GC **GCC T GTGCACAC = h**  **16** ACACAC **GTGCCAC T GGGGATGGCCCCTGCCCC** G**T G**CC G **GTGCACAC = j**  **17** AC**CT**AC **GTGCCAC T GGGGATGGCCCCTGCCCC** ACACAC **GTGCACAC = k**  gcctgtgtgtggct next del 75 bp: *primer revRP* |


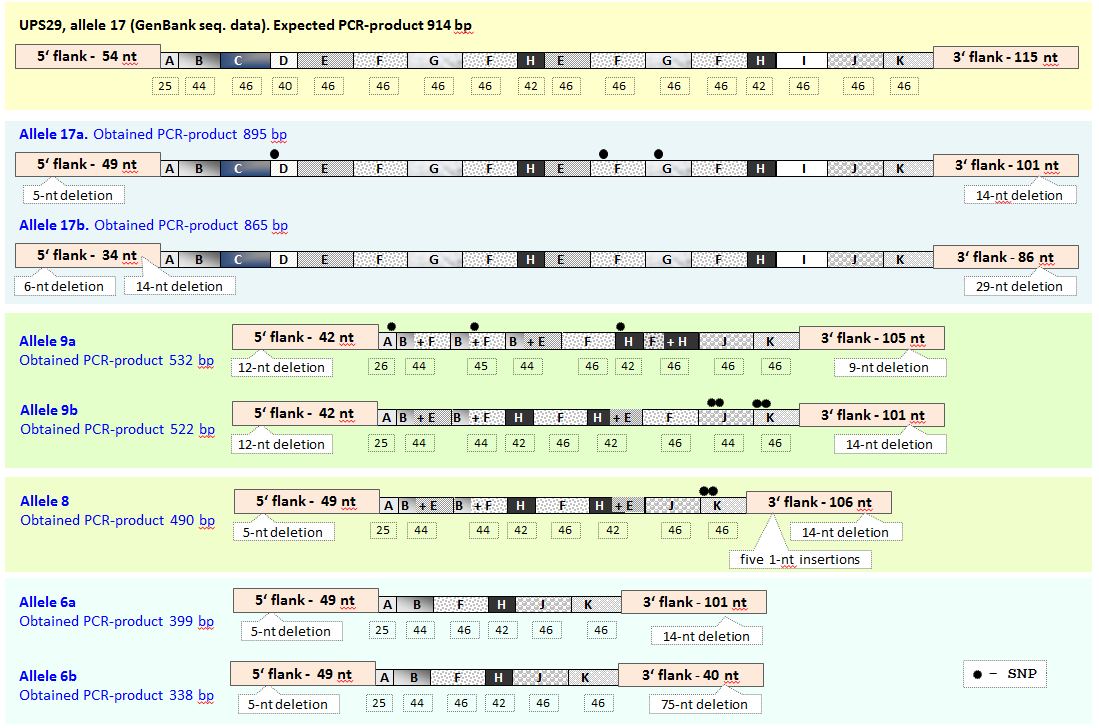


Figure S2. Schematic of UPS29 alleles according to sequencing results. Comparison with the reference sequence (AL096805, GeneID:116983; UPS29 allele of 17 repeats). Variants of UPS29 repeat units are indicated by rectangles with varying degrees of fill, their lengths are shown under repeats. Repeats “F+H” (46 nt), “B+E” (44 nt), “B+F” (44 nt) and “H+E” (42 nt) are “hybrid” (mixed) [Suchkova et al., 2007].


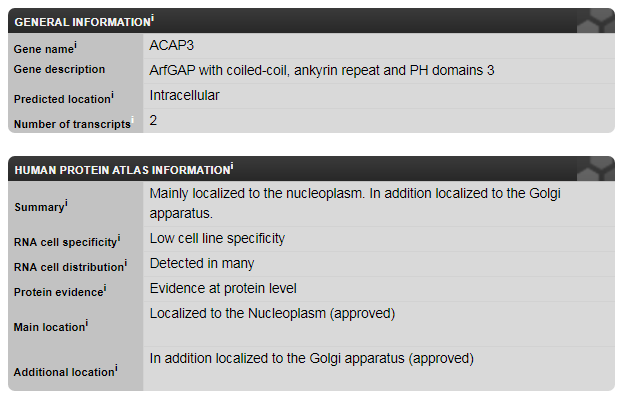

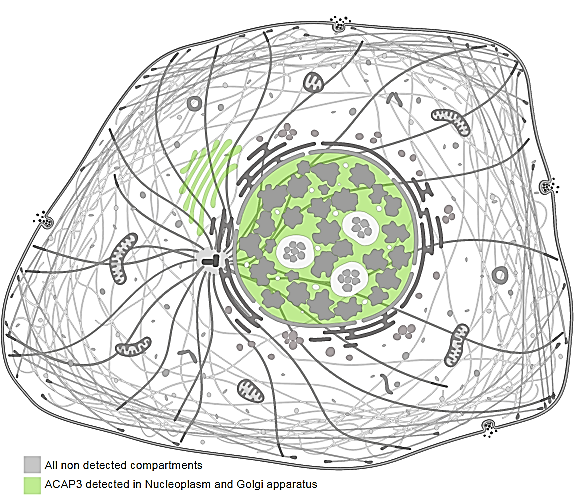

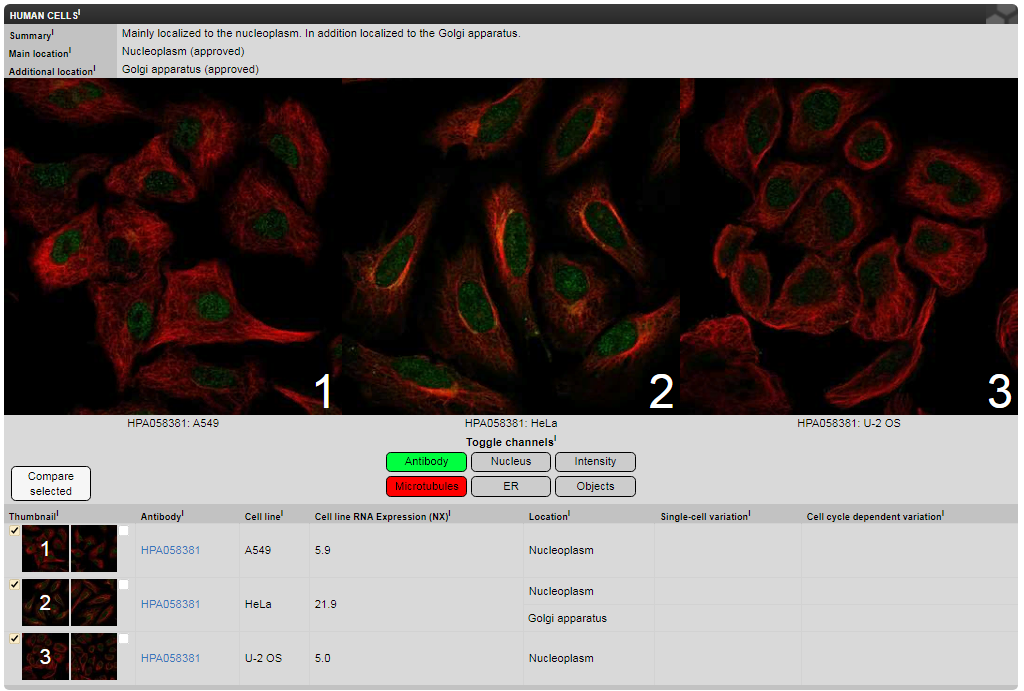


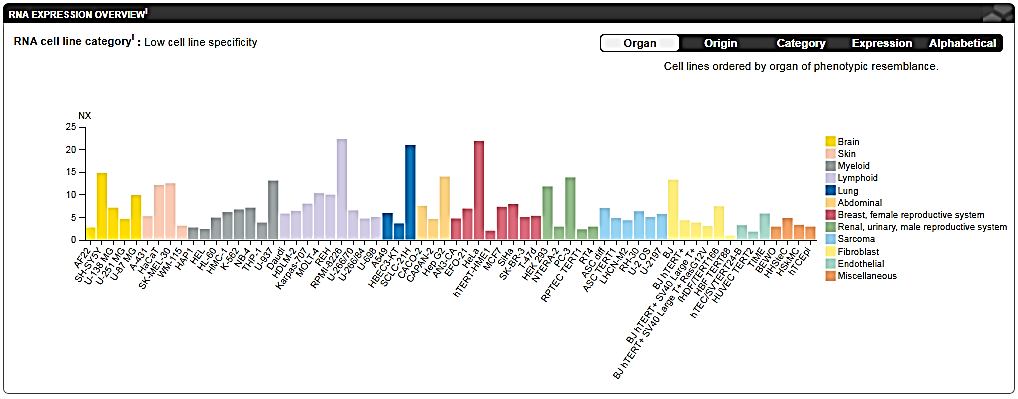


Figure S3. ACAP3 expression in cells. (The Human Protein Atlas, Cells, <https://www.proteinatlas.org/ENSG00000131584-ACAP3/cell> , accessed 21.05.2020).


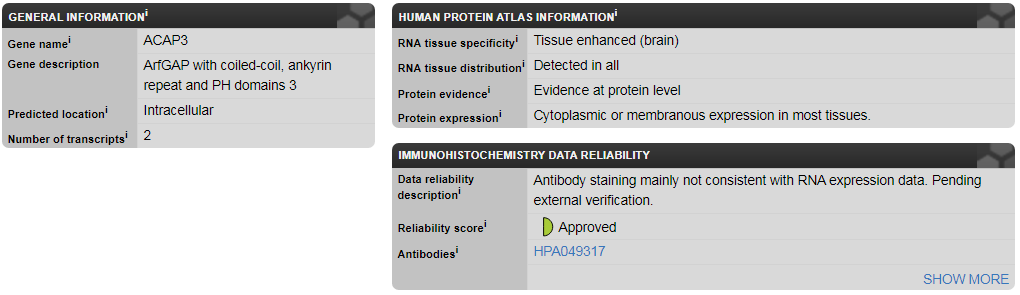


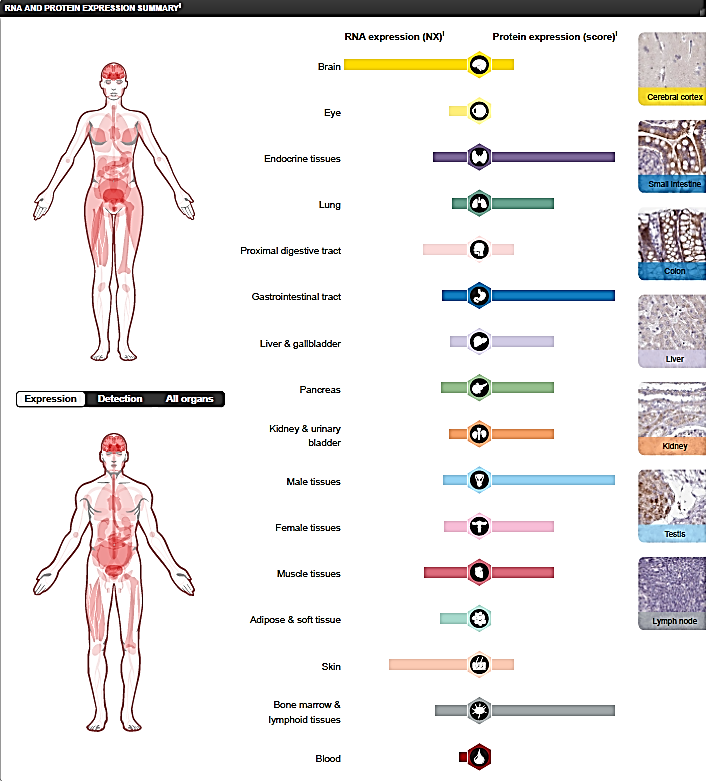


Figure S4. ACAP3 mRNA and protein expression in tissue (The Human Protein Atlas, Tissue, <https://www.proteinatlas.org/ENSG00000131584-ACAP3/tissue> , accessed 21.05.2020).


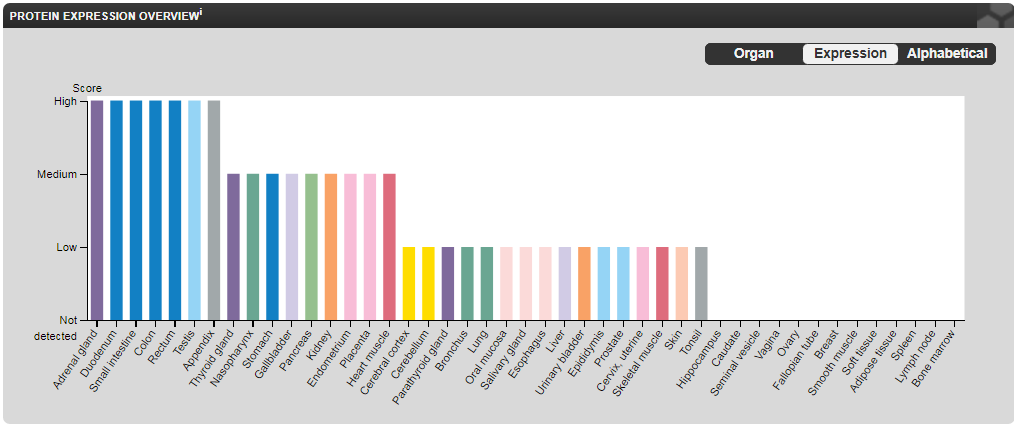


Figure S5. ACAP3 protein expression in tissue (The Human Protein Atlas, Tissue, <https://www.proteinatlas.org/ENSG00000131584-ACAP3/tissue> , accessed 21.05.2020).


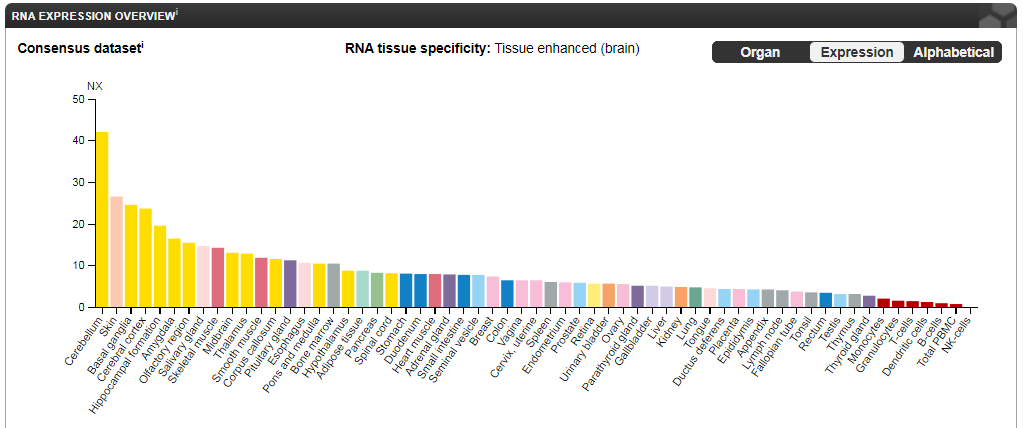


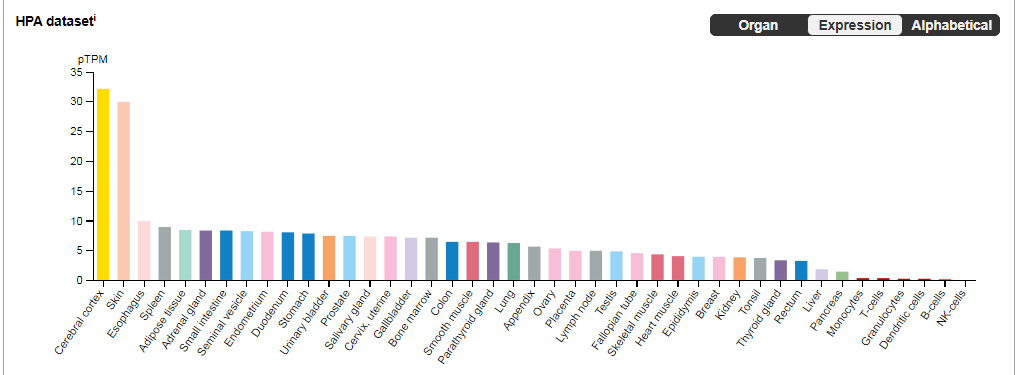


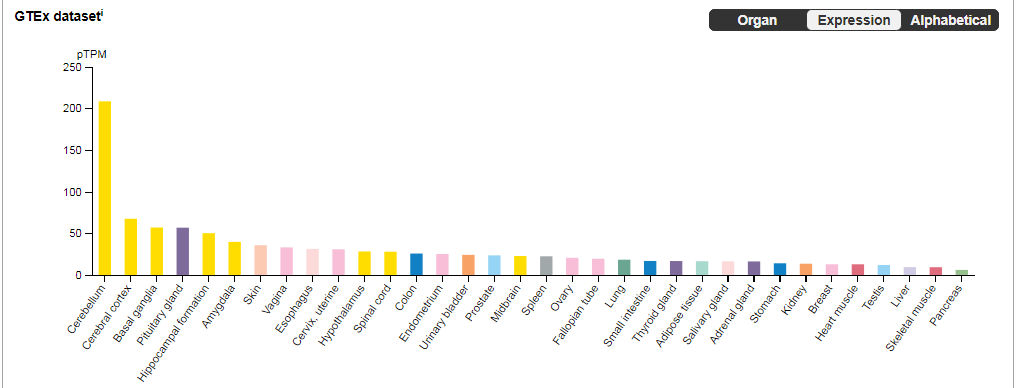


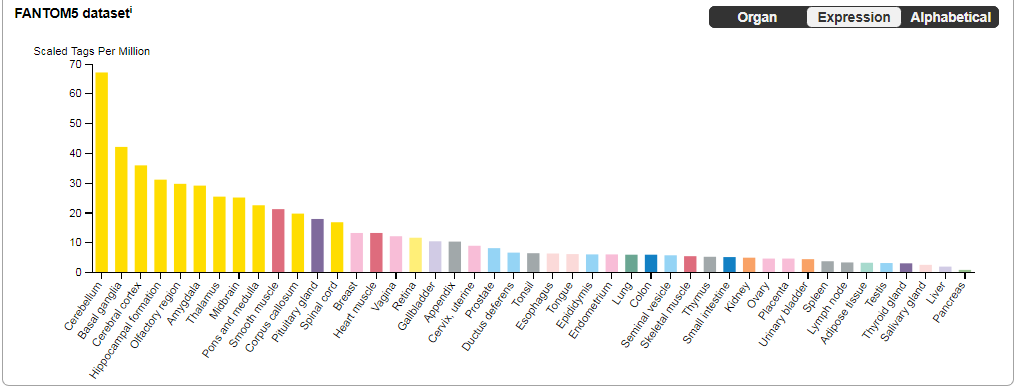


Figure S6. ACAP3 mRNA expression in tissue (The Human Protein Atlas, Tissue, <https://www.proteinatlas.org/ENSG00000131584-ACAP3/tissue> , accessed 21.05.2020).


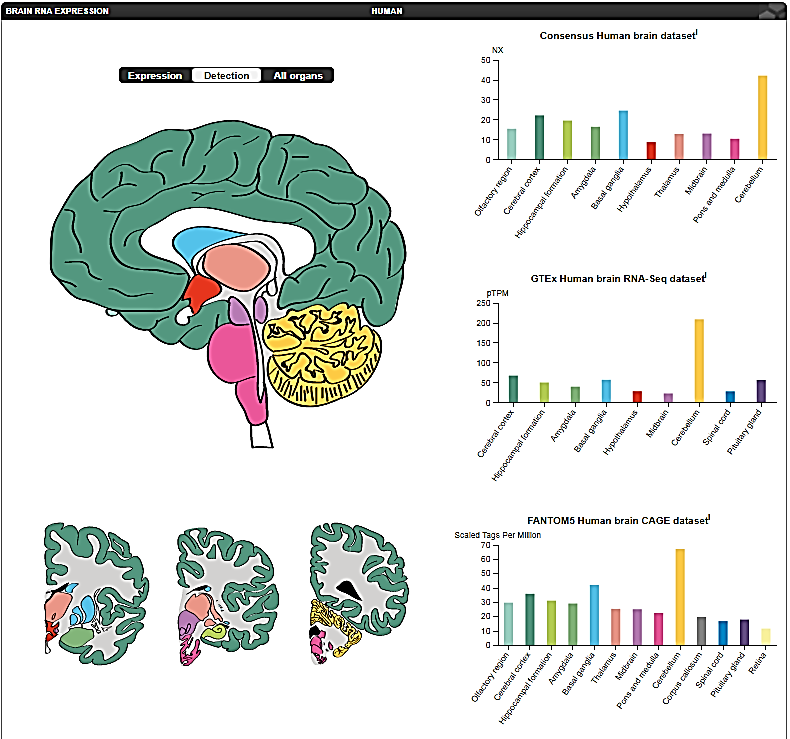


Figure S7. ACAP3 mRNA expression in brain (The Human Protein Atlas, Brain, <https://www.proteinatlas.org/ENSG00000131584-ACAP3/brain>, accessed 21.05.2020).


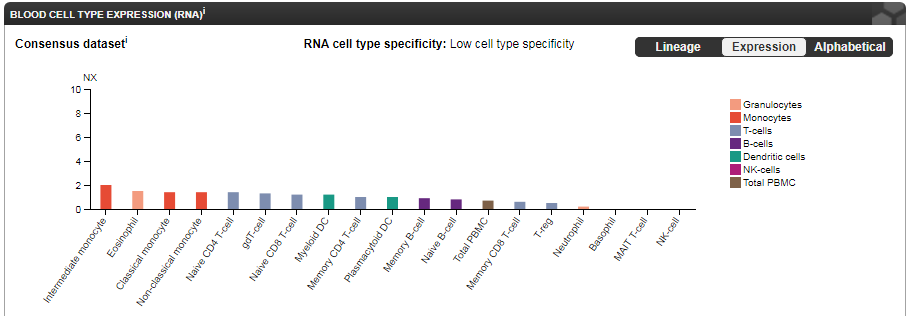


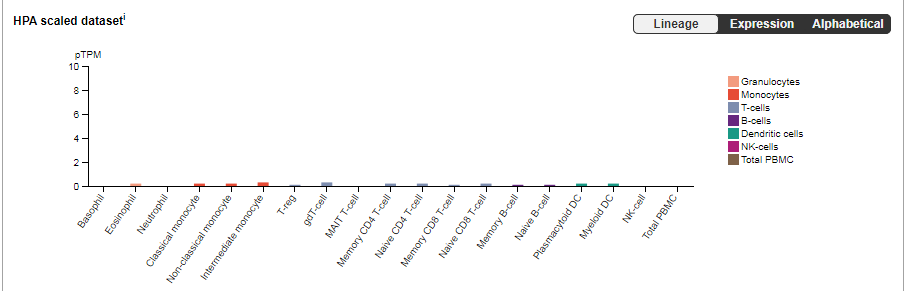


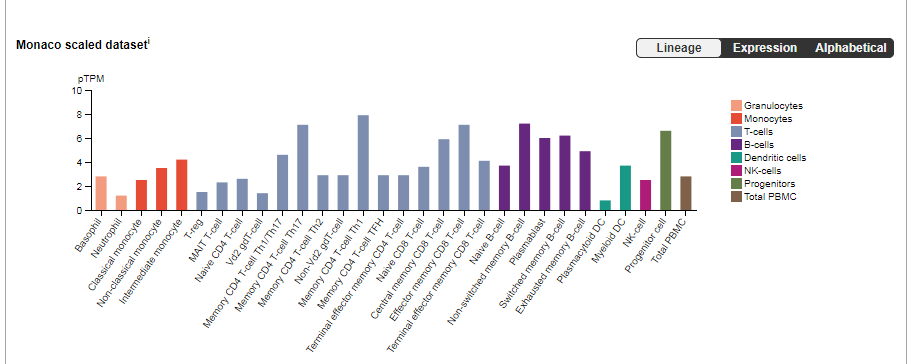


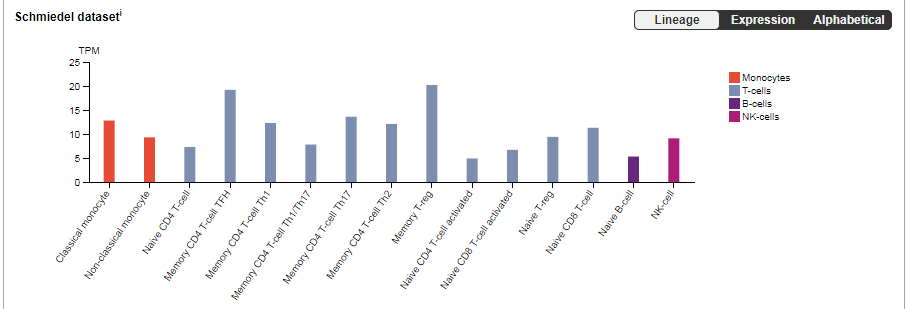


Figure S8. ACAP3 mRNA expression in blood cells (The Human Protein Atlas, Blood, https://www.proteinatlas.org/ENSG00000131584-ACAP3/blood, accessed 21.05.2020).

Table S4. ACAP3 protein structure (UniProt, <https://www.uniprot.org/uniprot/Q96P50>, accessed 08.06.2020).

| Protein | | | |
| --- | --- | --- | --- |
| Human ACAP3  UniProtKB -Q96P50 | | Arf-GAP with coiled-coil, ANK repeat and PH domain-containing protein 3 Length: 834 aa  Mass:92,495 Da | |
| Feature key | Position (s) | Description Actions | Length, aa |
| Domain | [268 – 363](https://www.uniprot.org/blast/?about=Q96P50%5b268-363%5d&key=Domain) | PH | 96 |
| Domain | [403 – 525](https://www.uniprot.org/blast/?about=Q96P50%5b403-525%5d&key=Domain) | Arf-GAP | 123 |
| Repeat | [702 – 731](https://www.uniprot.org/blast/?about=Q96P50%5b702-731%5d&key=Repeat) | ANK 1 | 30 |
| Repeat | [735 – 764](https://www.uniprot.org/blast/?about=Q96P50%5b735-764%5d&key=Repeat) | ANK 2 | 30 |
| Repeat | [768 – 797](https://www.uniprot.org/blast/?about=Q96P50%5b768-797%5d&key=Repeat) | ANK 3 | 30 |
| Zinc finger | [418 – 441](https://www.uniprot.org/blast/?about=Q96P50%5b418-441%5d&key=Zinc%20finger) | C4-type | 24 |
| 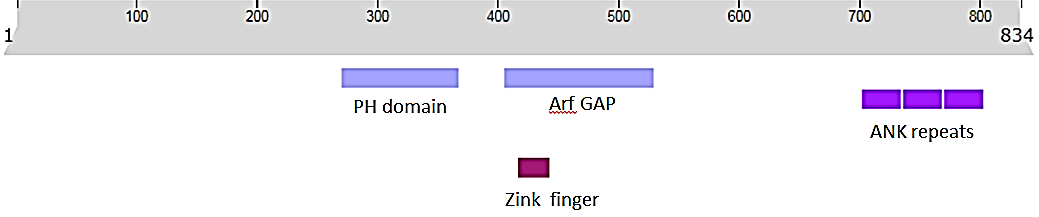 | | | |

Table S5. Some human genes from 1p36-p32, the products of which involve in the formation, functioning and pathological changes of the nervous and cardiovascular systems (Atlas of Genetics and Cytogenetics in Oncology and Haematology, <http://atlasgeneticsoncology.org/Indexbychrom/idxg_1.html> , accessed 22.05.2020; GeneCards. The Human Gene Database, <https://www.genecards.org/> , accessed 08.06.2020).

| **Symbol** | **GoldenPath hg38 (Mb) (↓)** | **Location (↓)** | **Description** |
| --- | --- | --- | --- |
| [TTLL10](http://atlasgeneticsoncology.org/Genes/GC_TTLL10.html) | [1179.697](http://genome.ucsc.edu/cgi-bin/hgTracks?position=chr1:1179697-1185863) | 1p36.33 | tubulin tyrosine ligase like 10 ( Cone-Rod Dystrophy 16, Achromatopsia) |
| [SDF4](http://atlasgeneticsoncology.org/Genes/GC_SDF4.html) | [1216.908](http://genome.ucsc.edu/cgi-bin/hgTracks?position=chr1:1216908-1232067) | 1p36.33 | stromal cell derived factor 4 (it may be involved in regulating **calcium** **dependent cellular activities** ) |
| [**UBE2J2**](http://atlasgeneticsoncology.org/Genes/GC_UBE2J2.html) | [1253.912](http://genome.ucsc.edu/cgi-bin/hgTracks?position=chr1:1253912-1273854) | 1p36.33 | ubiquitin conjugating enzyme E2 J2 (Breast, **adenocarcinoma**) |
| [**SCNN1D**](http://atlasgeneticsoncology.org/Genes/GC_SCNN1D.html) | [1280.436](http://genome.ucsc.edu/cgi-bin/hgTracks?position=chr1:1280436-1292029) | 1p36.33 | sodium channel epithelial 1 subunit delta (**Cerebrovascular pathologies**, structural changes in the brain, as well as the development of **neuroblastoma**  ?) |
| [**ACAP3**](http://atlasgeneticsoncology.org/Genes/GC_ACAP3.html) | [1292.384](http://genome.ucsc.edu/cgi-bin/hgTracks?position=chr1:1292384-1307889) | 1p36.33 | ArfGAP with coiled-coil, ankyrin repeat and PH domains 3 |
| [MIR6726](http://atlasgeneticsoncology.org/Genes/GC_MIR6726.html) | [1296.110](http://genome.ucsc.edu/cgi-bin/hgTracks?position=chr1:1296110-1296170) | 1p36.33 | microRNA 6726 |
| [SNORD167](http://atlasgeneticsoncology.org/Genes/GC_SNORD167.html) | [1304.729](http://genome.ucsc.edu/cgi-bin/hgTracks?position=chr1:1304729-1304812) | 1p36.33 | small nucleolar RNA, C/D box 167 |
| [PUSL1](http://atlasgeneticsoncology.org/Genes/GC_PUSL1.html) | [1308.580](http://genome.ucsc.edu/cgi-bin/hgTracks?position=chr1:1308580-1311677) | 1p36.33 | pseudouridine synthase like 1 |
| [INTS11](http://atlasgeneticsoncology.org/Genes/GC_INTS11.html) | [1311.585](http://genome.ucsc.edu/cgi-bin/hgTracks?position=chr1:1311585-1324687) | 1p36.33 | integrator complex subunit 11 ( Meningococcal **Meningitis** and Factor Viii Deficiency) |
| [TAS1R3](http://atlasgeneticsoncology.org/Genes/GC_TAS1R3.html) | [1331.346](http://genome.ucsc.edu/cgi-bin/hgTracks?position=chr1:1331346-1335320) | 1p36.33 | taste 1 receptor member 3 (G protein-coupled receptor activity and taste receptor activity.) |
| [DVL1](http://atlasgeneticsoncology.org/Genes/DVL1ID463ch1p36.html) | [1335.278](http://genome.ucsc.edu/cgi-bin/hgTracks?position=chr1:1335278-1349112) | 1p36.33 | dishevelled segment polarity protein 1 (**Neuroblastoma,** Cardiomyocyte hypertrophy and ERK Signaling. ) |
| [AURKAIP1](http://atlasgeneticsoncology.org/Genes/GC_AURKAIP1.html) | [1373.730](http://genome.ucsc.edu/cgi-bin/hgTracks?position=chr1:1373730-1375438) | 1p36.33 | aurora kinase A interacting protein 1 |
| [ATAD3C](http://atlasgeneticsoncology.org/Genes/GC_ATAD3C.html) | [1449.689](http://genome.ucsc.edu/cgi-bin/hgTracks?position=chr1:1449689-1470158) | 1p36.33 | ATPase family AAA domain containing 3C  **Pontocerebellar Hypoplasia,**  **Type 4, Pontocerebellar Hypoplasia, Type 3** |
| [ATAD3B](http://atlasgeneticsoncology.org/Genes/GC_ATAD3B.html) | [1471.755](http://genome.ucsc.edu/cgi-bin/hgTracks?position=chr1:1471755-1496204) | 1p36.33 | ATPase family AAA domain containing 3B **Pontocerebellar Hypoplasia,**  **Type 4, Pontocerebellar Hypoplasia, Type 3** |
| [ATAD3A](http://atlasgeneticsoncology.org/Genes/GC_ATAD3A.html) | [1512.143](http://genome.ucsc.edu/cgi-bin/hgTracks?position=chr1:1512143-1534687) | 1p36.33 | ATPase family AAA domain containing 3A **Pontocerebellar Hypoplasia,**  **Type 4, Pontocerebellar Hypoplasia, Type 3** |
| [MMP23B](http://atlasgeneticsoncology.org/Genes/GC_MMP23B.html) | [1632.181](http://genome.ucsc.edu/cgi-bin/hgTracks?position=chr1:1632181-1634654) | 1p36.33 | matrix metallopeptidase 23B (**Neuroblastoma, Chromosome** **1p36 Deletion Syndrome**) |
| [MMP23A](http://atlasgeneticsoncology.org/Genes/GC_MMP23A.html) | [1632.780](http://genome.ucsc.edu/cgi-bin/hgTracks?position=chr1:1632780-1634651) | 1p36.33 | matrix metallopeptidase 23A (pseudogene) (**Neuroblastoma, Chromosome** **1p36 Deletion Syndrome**) |
| [CDK11B](http://atlasgeneticsoncology.org/Genes/GC_CDK11B.html) (Cdc2L1-2) | [1635.226](http://genome.ucsc.edu/cgi-bin/hgTracks?position=chr1:1635226-1659097) | 1p36.33 | cyclin dependent kinase 11B (**Neuroblastoma, Chromosome** **1p36 Deletion Syndrome**) |
| D1Z2 |  | 1p36.33 | genetic marker containing a highly polymorphic Variable number tandem repeat consisting of 40-nt repeats (**Neuroblastoma, Chromosome** **1p36 Deletion Syndrome**)  [Gururaian et al., 1990] |
| [GNB1](http://atlasgeneticsoncology.org/Genes/GC_GNB1.html) | [1785.285](http://genome.ucsc.edu/cgi-bin/hgTracks?position=chr1:1785285-1891117) | 1p36.33 | G protein subunit beta 1 (**Mental Retardation**, Autosomal Dominant 42, Hypotonia) |
| [CALML6](http://atlasgeneticsoncology.org/Genes/GC_CALML6.html) | [1914.827](http://genome.ucsc.edu/cgi-bin/hgTracks?position=chr1:1914827-1917294) | 1p36.33 | calmodulin like 6 (Conjunctivochalasis , **Chromosome** **1p36 Deletion Syndrome, Mental Retardation**) |
| [GABRD](http://atlasgeneticsoncology.org/Genes/GC_GABRD.html) | [2019.329](http://genome.ucsc.edu/cgi-bin/hgTracks?position=chr1:2019329-2030753) | 1p36.33 | gamma-aminobutyric acid type A receptor subunit delta (**Epilepsy**, idiopathic generalized 10) |
| [PEX10](http://atlasgeneticsoncology.org/Genes/GC_PEX10.html) | [2404.802](http://genome.ucsc.edu/cgi-bin/hgTracks?position=chr1:2404802-2412571) | 1p36.32 | peroxisomal biogenesis factor 10 (**Mental retardation**) |
| [PLCH2](http://atlasgeneticsoncology.org/Genes/GC_PLCH2.html) | [2476.315](http://genome.ucsc.edu/cgi-bin/hgTracks?position=chr1:2476315-2505530) | 1p36.32 | phospholipase C eta 2, contained minisatellite D1S80 (**Neuroblastoma ?**) |
| [PANK4](http://atlasgeneticsoncology.org/Genes/GC_PANK4.html) | [2508.531](http://genome.ucsc.edu/cgi-bin/hgTracks?position=chr1:2508531-2526596) | 1p36.32 | pantothenate kinase 4 (inactive) (**Neurodegeneration**) |
| [TPRG1L](http://atlasgeneticsoncology.org/Genes/GC_TPRG1L.html) | [3624.992](http://genome.ucsc.edu/cgi-bin/hgTracks?position=chr1:3624992-3630130) | 1p36.32 | tumor protein p63 regulated 1 like |
| [WRAP73](http://atlasgeneticsoncology.org/Genes/GC_WRAP73.html) | [3630.767](http://genome.ucsc.edu/cgi-bin/hgTracks?position=chr1:3630767-3650107) | 1p36.32 | WD repeat containing, antisense to TP73 **Neuroblastoma** |
| [TP73](http://atlasgeneticsoncology.org/Genes/GC_TP73.html) (NB) | [3652.565](http://genome.ucsc.edu/cgi-bin/hgTracks?position=chr1:3652565-3736201) | 1p36.32 | tumor protein p73 (**Neuroblastoma, Alzheimer's disease**?) |
| [CCDC27](http://atlasgeneticsoncology.org/Genes/GC_CCDC27.html) | [3752.401](http://genome.ucsc.edu/cgi-bin/hgTracks?position=chr1:3752401-3771645) | 1p36.32 | coiled-coil domain containing 27 |
| [SMIM1](http://atlasgeneticsoncology.org/Genes/GC_SMIM1.html) | [3772.761](http://genome.ucsc.edu/cgi-bin/hgTracks?position=chr1:3772761-3775982) | 1p36.32 | small integral membrane protein 1 (Vel blood group) |
| [LRRC47](http://atlasgeneticsoncology.org/Genes/GC_LRRC47.html) | [3780.220](http://genome.ucsc.edu/cgi-bin/hgTracks?position=chr1:3780220-3796504) | 1p36.32 | leucine rich repeat containing 47 |
| [CEP104](http://atlasgeneticsoncology.org/Genes/GC_CEP104.html) | [3812.081](http://genome.ucsc.edu/cgi-bin/hgTracks?position=chr1:3812081-3857233) | 1p36.32 | centrosomal protein 104 |
| [DFFB](http://atlasgeneticsoncology.org/Genes/GC_DFFB.html) | [3857.267](http://genome.ucsc.edu/cgi-bin/hgTracks?position=chr1:3857267-3885429) | 1p36.32 | DNA fragmentation factor subunit beta (**Temporal lobe epilepsy**) |
| [KCNAB2](http://atlasgeneticsoncology.org/Genes/GC_KCNAB2.html) | [6026.013](http://genome.ucsc.edu/cgi-bin/hgTracks?position=chr1:6026013-6101193) | 1p36.31 | potassium voltage-gated channel subfamily A regulatory beta subunit 2 |
| [**CHD5**](http://atlasgeneticsoncology.org/Genes/CHD5ID44521ch1p36.html) | [6101.787](http://genome.ucsc.edu/cgi-bin/hgTracks?position=chr1:6101787-6180134) | 1p36.31 | chromodomain helicase DNA binding protein 5 (Neuron-specific protein that may function in chromatin remodeling and gene transcription; **Neuroblastoma**) |
| [ACOT7](http://atlasgeneticsoncology.org/Genes/GC_ACOT7.html) | [6264.272](http://genome.ucsc.edu/cgi-bin/hgTracks?position=chr1:6264272-6358944) | 1p36.31 | acyl-CoA thioesterase 7 (**Mesial temporal lobe epilepsy**) |
| [HES2](http://atlasgeneticsoncology.org/Genes/GC_HES2.html) | [6415.234](http://genome.ucsc.edu/cgi-bin/hgTracks?position=chr1:6415234-6419919) | 1p36.31 | hes family bHLH transcription factor 2 |
| [ESPN](http://atlasgeneticsoncology.org/Genes/GC_ESPN.html) | [6424.788](http://genome.ucsc.edu/cgi-bin/hgTracks?position=chr1:6424788-6460944) | 1p36.31 | Espin (Autosomal recessive neurosensory deafness, and autosomal dominant sensorineural deafness without vestibular involvement) |
| [PLEKHG5](http://atlasgeneticsoncology.org/Genes/GC_PLEKHG5.html) | [6466.092](http://genome.ucsc.edu/cgi-bin/hgTracks?position=chr1:6466092-6520061) | 1p36.31 | pleckstrin homology and RhoGEF domain containing G5 |
| [PARK7](http://atlasgeneticsoncology.org/Genes/PARK7ID41639ch1p36.html) (DJ-1) | [7961.654](http://genome.ucsc.edu/cgi-bin/hgTracks?position=chr1:7961654-7985282) | 1p36.23 | **Parkinsonism** associated deglycase |
| [ENO1](http://atlasgeneticsoncology.org/Genes/ENO1ID40453ch1p36.html) | [8861.002](http://genome.ucsc.edu/cgi-bin/hgTracks?position=chr1:8861002-8878686) | 1p36.23 | enolase 1 ( **Hashimoto Encephalopathy** and Cancer-Associated Retinopathy) |
| [ENO1-AS1](http://atlasgeneticsoncology.org/Genes/GC_ENO1-AS1.html) | [8878.835](http://genome.ucsc.edu/cgi-bin/hgTracks?position=chr1:8878835-8879884) | 1p36.23 | ENO1 antisense RNA 1 |
| [UBE4B](http://atlasgeneticsoncology.org/Genes/GC_UBE4B.html) | [10032.983](http://genome.ucsc.edu/cgi-bin/hgTracks?position=chr1:10032983-10181238) | 1p36.22 | ubiquitination factor E4B (**Neurodegenetation**) |
| [CASZ1](http://atlasgeneticsoncology.org/Genes/CASZ1ID45989ch1p36.html) | [10647.206](http://genome.ucsc.edu/cgi-bin/hgTracks?position=chr1:10647206-10796676) | 1p36.22 | castor zinc finger 1 (Stroke) |
| [MTHFR](http://atlasgeneticsoncology.org/Genes/MTHFRID41448ch1p36.html) | [11785.730](http://genome.ucsc.edu/cgi-bin/hgTracks?position=chr1:11785730-11806103) | 1p36.22 | methylenetetrahydrofolate reductase (It influences susceptibility to occlusive **vascular disease**, **neural tube defects**, colon cancer and acute leukemia) |
| [CLCN6](http://atlasgeneticsoncology.org/Genes/GC_CLCN6.html) | [11806.096](http://genome.ucsc.edu/cgi-bin/hgTracks?position=chr1:11806096-11843144) | 1p36.22 | chloride voltage-gated channel 6 |
| [HNRNPCL1](http://atlasgeneticsoncology.org/Genes/GC_HNRNPCL1.html) | [12847.377](http://genome.ucsc.edu/cgi-bin/hgTracks?position=chr1:12847377-12848756) | 1p36.21 | heterogeneous nuclear ribonucleoprotein C like 1 (Intellectual disability and **seizures** ?) |
| [HNRNPCL3](http://atlasgeneticsoncology.org/Genes/GC_HNRNPCL3.html) | [13061.232](http://genome.ucsc.edu/cgi-bin/hgTracks?position=chr1:13061232-13062237) | 1p36.21 | heterogeneous nuclear ribonucleoprotein C like 3 (Intellectual disability and **seizures** ?) |
| [HNRNPCL4](http://atlasgeneticsoncology.org/Genes/GC_HNRNPCL4.html) | [13061.310](http://genome.ucsc.edu/cgi-bin/hgTracks?position=chr1:13061310-13062237) | 1p36.21 | heterogeneous nuclear ribonucleoprotein C like 4 (Intellectual disability and **seizures** ?) |
| [**CASP9**](http://atlasgeneticsoncology.org/Genes/CASP9ID423ch1p36.html) | [15491.401](http://genome.ucsc.edu/cgi-bin/hgTracks?position=chr1:15491401-15524790) | 1p36.21 | caspase 9 (cell apoptosis) |
| [DNAJC16](http://atlasgeneticsoncology.org/Genes/GC_DNAJC16.html) | [15526.813](http://genome.ucsc.edu/cgi-bin/hgTracks?position=chr1:15526813-15571733) | 1p36.21 | DnaJ heat shock protein family (Hsp40) member C16 |
| [SRARP](http://atlasgeneticsoncology.org/Genes/GC_SRARP.html) | [16004.236](http://genome.ucsc.edu/cgi-bin/hgTracks?position=chr1:16004236-16006695) | 1p36.13 | steroid receptor associated and regulated protein |
| [HSPB7](http://atlasgeneticsoncology.org/Genes/GC_HSPB7.html) | [16014.028](http://genome.ucsc.edu/cgi-bin/hgTracks?position=chr1:16014028-16018790) | 1p36.13 | heat shock protein family B (small) member 7 |
| [CLCNKA](http://atlasgeneticsoncology.org/Genes/GC_CLCNKA.html) | [16021.991](http://genome.ucsc.edu/cgi-bin/hgTracks?position=chr1:16021991-16034050) | 1p36.13 | chloride voltage-gated channel Ka |
| [CLCNKB](http://atlasgeneticsoncology.org/Genes/GC_CLCNKB.html) | [16048.762](http://genome.ucsc.edu/cgi-bin/hgTracks?position=chr1:16048762-16057326) | 1p36.13 | chloride voltage-gated channel Kb |
| [NBPF1](http://atlasgeneticsoncology.org/Genes/GC_NBPF1.html) | [16562.427](http://genome.ucsc.edu/cgi-bin/hgTracks?position=chr1:16562427-16613605) | 1p36.13 | NBPF member 1 (**Neuroblastoma**) |
| [ESPNP](http://atlasgeneticsoncology.org/Genes/GC_ESPNP.html) | [16691.218](http://genome.ucsc.edu/cgi-bin/hgTracks?position=chr1:16691218-16720157) | 1p36.13 | espin pseudogene |
| [ATP13A2](http://atlasgeneticsoncology.org/Genes/GC_ATP13A2.html) (PARK9) | [16985.958](http://genome.ucsc.edu/cgi-bin/hgTracks?position=chr1:16985958-17011972) | 1p36.13 | ATPase cation transporting 13A2 (**Kufor-Rakeb syndrome**/ **Parkinson disease** 9) |
| [UBR4](http://atlasgeneticsoncology.org/Genes/GC_UBR4.html) | [19074.506](http://genome.ucsc.edu/cgi-bin/hgTracks?position=chr1:19074506-19210252) | 1p36.13 | ubiquitin protein ligase E3 component n-recognin 4 (**Stroke** ) |
| [NBL1](http://atlasgeneticsoncology.org/Genes/GC_NBL1.html) | [19643.229](http://genome.ucsc.edu/cgi-bin/hgTracks?position=chr1:19643229-19658456) | 1p36.13 | NBL1, DAN family BMP antagonist (**Neuroblastoma** ?) |
| [HTR6](http://atlasgeneticsoncology.org/Genes/GC_HTR6.html) | [19665.287](http://genome.ucsc.edu/cgi-bin/hgTracks?position=chr1:19665287-19680966) | 1p36.13 | 5-hydroxytryptamine receptor 6 (Serotonin receptor) |
| [CAMK2N1](http://atlasgeneticsoncology.org/Genes/GC_CAMK2N1.html) | [20482.391](http://genome.ucsc.edu/cgi-bin/hgTracks?position=chr1:20482391-20486235) | 1p36.12 | calcium/calmodulin dependent protein kinase II inhibitor 1 |
| [PINK1](http://atlasgeneticsoncology.org/Genes/GC_PINK1.html) (PARK6) | [20633.455](http://genome.ucsc.edu/cgi-bin/hgTracks?position=chr1:20633455-20651511) | 1p36.12 | PTEN induced kinase 1 (Autosomal recessive early-onset **Parkinson disease**) |
| [EPHB2](http://atlasgeneticsoncology.org/Genes/GC_EPHB2.html) | [22710.838](http://genome.ucsc.edu/cgi-bin/hgTracks?position=chr1:22710838-22921500) | 1p36.12 | EPH receptor B2 (**Brain cancer** susceptibility, **Angiogenesis**, Synaptic interactions) |
| [HTR1D](http://atlasgeneticsoncology.org/Genes/GC_HTR1D.html) | [23191.895](http://genome.ucsc.edu/cgi-bin/hgTracks?position=chr1:23191895-23194729) | 1p36.12 | 5-hydroxytryptamine receptor 1D (Serotonin receptor) |
| [HNRNPR](http://atlasgeneticsoncology.org/Genes/GC_HNRNPR.html) | [23304.690](http://genome.ucsc.edu/cgi-bin/hgTracks?position=chr1:23304690-23344364) | 1p36.12 | heterogeneous nuclear ribonucleoprotein R (Intellectual disability and **seizures** ?) |
| [SYTL1](http://atlasgeneticsoncology.org/Genes/GC_SYTL1.html) | [27341.992](http://genome.ucsc.edu/cgi-bin/hgTracks?position=chr1:27341992-27353932) | 1p36.11 | synaptotagmin like 1 (**Epilepsy** and lamotrigine-induced maculopapular eruptions ?) |
| [DNAJC8](http://atlasgeneticsoncology.org/Genes/GC_DNAJC8.html) | [28200.279](http://genome.ucsc.edu/cgi-bin/hgTracks?position=chr1:28200279-28233031) | 1p35.3 | DnaJ heat shock protein family (Hsp40) member C8 |
| [C1orf94](http://atlasgeneticsoncology.org/Genes/GC_C1orf94.html) | [34166.883](http://genome.ucsc.edu/cgi-bin/hgTracks?position=chr1:34166883-34219130) | 1p35.1 | chromosome 1 open reading frame 94 (Generalized **epilepsy**) |
| [SLC2A1](http://atlasgeneticsoncology.org/Genes/GC_SLC2A1.html) | [42925.375](http://genome.ucsc.edu/cgi-bin/hgTracks?position=chr1:42925375-42959176) | 1p34.2 | solute carrier family 2 member 1 (**Epilepsy**, idiopathic generalized, susceptibility to, 12) |
| [SLC2A1-AS1](http://atlasgeneticsoncology.org/Genes/GC_SLC2A1-AS1.html) | [42959.049](http://genome.ucsc.edu/cgi-bin/hgTracks?position=chr1:42959049-42983358) | 1p34.2 | SLC2A1 antisense RNA 1 |
| [ACOT11](http://atlasgeneticsoncology.org/Genes/GC_ACOT11.html) | [54548.134](http://genome.ucsc.edu/cgi-bin/hgTracks?position=chr1:54548134-54610332) | 1p32.3 | acyl-CoA thioesterase 11 (**Epilepsy** ?) |
| [USP24](http://atlasgeneticsoncology.org/Genes/GC_USP24.html) (PARK10) | [55066.359](http://genome.ucsc.edu/cgi-bin/hgTracks?position=chr1:55066359-55215366) | 1p32.3 | ubiquitin specific peptidase 24 (**Parkinson's disease** or age at onset of disease) (AAOPD) |
| FRA1A | GRCh38/hg38 unknown strand | 1p36 | **fragile site,** aphidicolin type, common, fra(1)(p36) is an uncategorized gene |
| DYT13 |  | 1p36.13-36.32 | Dystonia 13, Torsion, Autosomal Dominant and Dystonia. |
| SCA24, SCASI |  | 1p36 | Spinocerebellar Ataxia With Saccadic Intrusions |

**Sp1 (801)**

#### AML-1a

NF-Atp

**Sp-1**

CAC-bind pr

Ttk

NF-1

HNF-3

MTF1

MBF I

CP-2

CR2 (850)

HES-1

MZF-1

USF

**muEBP-C2**

**TFE3-S**

**USE**

**SREP**

**USF2**

**GR**

HNF

MBF I

MycCF1

D1

HNF-4α1

HNF-4α2

HNF-4α4

HNF-4α7

HNF-4α3

HNF-4γ

HNF-4β

**Sp-1** (754)

CAC-bi..pr

NF-E2

**Sp-1 (759)**

##### AML-1c

### AML –1a

AP-1 (767)

AP-4

CCK-1a

CG-1

c-Myc

###### CREB

Max1

USF

TDEF

Twi

**MBFI** (771)

MTFI

NF-Atp

Ttk-69K (789)

D1

HNF-4α1

HNF-4α2

HNF-4α4

HNF-4α7

HNF-4α3

HNF-4γ

HNF-4β

**Sp-1** (700)

CAC-bind..pr

NF-E2

# C/EBP

MBF-I

MTF-1

HNF-3

Ap-2 (741)

Ap2αA

Ap2αB

7

D1

HNF-4α1

HNF-4α2

HNF-4α4

HNF-4α7

HNF-4α3

HNF-4γ

HNF-4β

**Sp-1** (660)

CAC-bind..pr

NF-E2

GCF (667)

## Sp-1 BTEB

MAZ

TBP

TFIID

AP2

MZF1

ZF5

# C/EBP (677)

MBF-I

MTF-1

HNF-3

**n-Myc**

GCF

PHO4

AP-1 (689)

AP-4

CCK-1a

CG-1

c-Myc

###### CREB

Max1

USF

TDEF

Twi

**-**

**Ap2αB** (699)

**Ap2αA**

D1

HNF-4α1

HNF-4α2

HNF-4α4

HNF-4α7

HNF-4α3

HNF-4γ

HNF-4β

**Sp-1** (614)

CAC-bind..pr

NF-E2

GCF (621)

## Sp-1 BTEB

MAZ

TBP

TFIID

AP2 (623)

MZF1

ZF5

# C/EBP (631)

MBF-I

MTF-1

HNF-3

**n-Myc**

GCF

PHO4

AP-1

AP-4

CCK-1a

CG-1

c-Myc

###### CREB

Max1

USF

TDEF

Twi

Ap-2 (649)

Ap2αB

Ap2αA

D1

HNF-4α1

HNF-4α2

HNF-4α4

HNF-4α7

HNF-4α3

HNF-4γ

HNF-4β

**Sp-1** (572)

CAC-bind..pr

NF-E2

# C/EBP

MBF-I

MTF-1

HNF-3

**muEBP-C2**

**TFE3-S** (595)

**USE**

**SREP**

**USF2**

**GR** (599)

Ap-2 (600)

Ap2αB

Ap2αA

D1

HNF-4α1

HNF-4α2

HNF-4α4

HNF-4α7

HNF-4α3

HNF-4γ

HNF-4β

**Sp-1** (526)

CAC-bind..pr

NF-E2

GCF (532)

## Sp-1 BTEB

MAZ

TBP

TFIID

AP2 (535)

MZF1

ZF5

# C/EBP

MBF-I

MTF-1

HNF-3

**n-Myc**

GCF

PHO4

AP-1 (555)

AP-4

CCK-1a

CG-1

c-Myc

###### CREB

Max1

USF

TDEF

Twi

Ap-2 (561)

Ap2αB

Ap2αA

D1

HNF-4α1

HNF-4α2

HNF-4α4

HNF-4α7

HNF-4α3

HNF-4γ

HNF-4β

**Sp-1** (482)

CAC-bind..pr

NF-E2

# C/EBP

MBF-I

MTF-1

HNF-3 (500)

**muEBP-C2**

**TFE3-S**

**USE**

**SREP**

**USF2**

**GR**

Ap-2 (515)

Ap2αB

Ap2αA

D1

HNF-4α1

HNF-4α2

HNF-4α4

HNF-4α7

HNF-4α3

HNF-4γ

HNF-4β

**Sp-1** (435)

CAC-bind..pr

NF-E2

# C/EBP

MBF-I

MTF-1

HNF-3

**n-Myc**

GCF

PHO4

AP-1 (463)

AP-4

CCK-1a

CG-1

c-Myc

###### CREB

Max1

USF

TDEF

Twi

Ap-2 (469)

Ap2αB

Ap2αA

# C/EBP (49)

MBF-I

MTF-1

HNF-3

**n-Myc**

GCF

PHO4

AP-1 (59)

AP-4

CCK-1a

CG-1

c-Myc

###### CREB

Max1

USF

TDEF

Twi

Ap-2 (65)

Ap2αB

Ap2αA

MBF-I

MTF-1

HNF-3

**n-Myc**

GCF

PHO4

AP-1 (145)

AP-4

CCK-1a

CG-1

c-Myc

###### CREB

Max1

USF

TDEF

Twi

Ap-2 (151)

Ap2αB

Ap2αA

D 1

HNF-4α1

HNF-4α2

HNF-4α4

HNF-4α7

HNF-4α3

HNF-4γ

HNF-4β

**Sp-1** (390)

CAC-bind..pr

NF-E2

GCF (394)

## Sp-1

## BTEB

MAZ

TBP

TFIID

AP2 (397)

MZF1

ZF5 (399)

# C/EBP

MBF-I

MTF-1

HNF-3

**n-Myc**

GCF

PHO4

AP-1 (417)

AP-4

CCK-1a

CG-1

c-Myc

###### CREB

Max1

USF

TDEF

Twi

Ap-2 (427)

Ap2αA

Ap2αB

D1 (71)

HNF-4α1 (75)

HNF-4α2

HNF-4α4

HNF-4α7

HNF-4α3

HNF-4γ

HNF-4β

**Sp-1** (78)

CAC-bind..pr

NF-E2

GCF

## Sp-1

## BTEB

MAZ

TBP

TFIID

AP2 (85)

MZF1

ZF5 (87)

# C/EBP (93)

MBF-I

MTF-1

HNF-3

**n-Myc**

GCF

PHO4

AP-1 (105)

AP-4

CCK-1a

CG-1

c-Myc

###### CREB

Max1

USF

TDEF

Twi

Ap-2 (111)

Ap2αB

Ap2αA

D1 (117)

HNF-4α1

HNF-4α2

HNF-4α4

HNF-4α7

HNF-4α3

HNF-4γ

HNF-4β

**Sp-1** (124)

CAC-bind..pr

NF-E2 (127)

D1 (157)

HNF-4α1

HNF-4α2

HNF-4α4

HNF-4α7

HNF-4α3

HNF-4γ

HNF-4β

**Sp-1** (164)

CAC-bind..pr

NF-E2 (167)

GCF

## Sp-1 (169)

#### BTEB

MAZ

TBP

TFIID

AP2 (171)

MZF1

ZF5 (173)

# C/EBP (179)

MBF-I

MTF-1

HNF-3

**n-Myc**

GCF

PHO4

AP-1 (191)

AP-4

CCK-1a

CG-1

c-Myc

###### CREB

Max1

USF

TDEF

Twi

Ap-2 (197)

Ap2αA (201)

Ap2αB

## D 1 (203)

HNF-4α1

HNF-4α2

HNF-4α4

HNF-4α7

HNF-4α3

HNF-4γ

HNF-4β

**Sp-1** (210)

CAC-bind..pr

NF-E2 (213)

# C/EBP (225)

MBF-I

MTF-1

HNF-3

**n-Myc**

GCF

PHO4

AP-1 (237)

AP-4

CCK-1a

CG-1

c-Myc

###### CREB

Max1

USF

TDEF

Twi

Ap-2 (243)

Ap2αB

Ap2αA

D1 (249)

HNF-4α1

HNF-4α2

HNF-4α4

HNF-4α7

HNF-4α3

HNF-4γ

HNF-4β

**Sp-1**

CAC-bind..pr

NF-E2 (259)

# C/EBP

MBF-I

MTF-1

HNF-3

**muEBP-C2**

**TFE3-S**

**USE**

**SREP**

**USF2**

**GR**

Ap-2

Ap2αB

Ap2αA

D1 (289)

HNF-4α1

HNF-4α2

HNF-4α4

HNF-4α7

HNF-4α3

HNF-4γ

HNF-4β

**Sp-1** (300)

CAC-bind..pr

NF-E2

GCF

## Sp-1 (307) BTEB

MAZ

TBP

TFIID

AP2 (309)

MZF1

ZF5 (311)

# C/EBP

MBF-I

MTF-1

HNF-3

**n-Myc**

GCF

PHO4

AP-1 (329)

AP-4

CCK-1a

CG-1

c-Myc

###### CREB

Max1

USF

TDEF

Twi

Ap-2 (335)

Ap2αB

Ap2αA

D1

HNF-4α1

HNF-4α2

HNF-4α4

HNF-4α7

HNF-4α3

HNF-4γ

HNF-4β

**Sp-1** (348)

CAC-bind..pr

NF-E2 (351)

# C/EBP (363)

MBF-I (365)

MTF-1

HNF-3

**muEBP-C2**

**TFE3-S**

**USE** (369)

**SREP**

**USF2**

**GR** (371)

Ap-2 (377)

Ap2αB

Ap2αA

1 31 46 160 201 384 427 610 653 790 895

**Part of exon14**

**Intron 14-15**

**5’ flank**

**a**

**c**

**b**

**d**

**e**

**f**

**g**

**f**

**h**

**h**

**e**

**g**

**f**

**f**

**3’ flank**

**i**

**k**

**j**

**UPS 29**

70 144 246 292 338 472 518 564 698 744

c-Myb

c-Myc

## Sp-1

## BTEB

Ttk

#### AML-1a

**Sp-1**

CAC-

binding

protein/

NF-E2

cacaca

cacaca

cacaca

Figure S9. Scheme of the location of potential sites of the interaction of transcription factors with UPS29.

Results *in silico* analysis of UPS29 (TESS. Transcription Element Search System, [www.cbil.upenn.edu/tess](http://www.cbil.upenn.edu/tess), accessed 2008) [Shubina et al., 2009]. The numbers under the schematic representation of the PCR fragment of UPS29 (allele of 17 repeats) indicate the ordinal numbers of nucleotides in UPS29. The numbers in parentheses indicate the ordinal numbers of nucleotides in UPS29, where the noted transcription factors can theoretically bind with UPS29. We would like to emphasize the following transcription factors, the disruption of the binding of which with UPS29 may be one of the explanations of the mechanisms of the revealed association of this minisatellite with epilepsy and Parkinson's disease: n-Myc (it involve in control of proliferation and differentiation), AML-1c (it associate with the nuclear matrix), BTEB (activator from tandem recognition elements, repressor from single sites; most abundant expression in brain), C/EBP (most abundant expression in brain), CREB (most abundant expression in brain), GCF (repressor at GC-rich sequences), MAZ (it may function in establishing active transcription complexes on the serotonin 1a receptor gene promoter), USF (it may have to direct stable preinitiation complex formation during chromosome assembly), SP1 (which can act in vivo by binding both to proximal promoter sequences and to remote enhancer sites and interacts with TFIID), USF (a transcriptional activator that regulates the formation of the stable preinitiation complex during nucleosome assembly, its binding to DNA depends on CpG methylation), TFIID (a TATA-box binding complex consisting of TBP and additional factors; interacts cooperatively with USF).

Table S6. High- and lower-confidence human imprinted gene candidates from 1p36.33-p36.32 (<https://www.geneimprint.com/site/genes-by-species>; <https://www.ncbi.nlm.nih.gov/protein/> ; <https://www.genecards.org/>) [Luedi et al., 2007; Luedi et al., supplement data, 2007 ].

| **Ensembl ID** | **Gene** | **Protein** | **Disease, function** | **Band** | **Predicted expressed allele** |
| --- | --- | --- | --- | --- | --- |
| ENSG00000131591 | *C1orf159* | Chromosome 1 Open Reading Frame 159 | Uncharacterized Protein C1orf159 | **1p36.33** | **Maternal** |
| ENSG00000184163 | *Q5EBL5*  *(Q5T7M4)*  *C1QTNF12* | C1q domain containing 2,  Adipolin, Adipose-derived insulin-sensitizing factor | Bleeding Disorder, Platelet-Type, 14.  Insulin-sensitizing adipocyte-secreted protein (adipokine) that regulates glucose metabolism in liver and adipose tissue. | **1p36.33** | **Maternal** |
| ENSG00000131584 | ***CENTB5 (ACAP3)*** | **Centaurin beta 5,**  Arf-GAP with coiled-coil, ANK repeat and PH domain-containing protein 3 | Endocytosis, GTPase activator activity*,* GTPase-activating protein for the ADP ribosylation factor family | **1p36.33** | **Maternal** |
| ENSG00000127054 | *INTS11* | Integrator Complex Subunit 11 | Meningococcal Meningitis,  Factor Viii Deficiency. | **1p36.33** | **Maternal** |
| ENSG00000169962 | *TAS1R3* | Taste 1 receptor member 3 | Dental caries. Signaling by GPCR, Sweet Taste Signaling. | **1p36.33** | **Maternal** |
| ENSG00000107404 | *DVL* | Dishevelled segment polarity protein 1 | Candidate gene for neuroblastomatous transformation. It regulates cell proliferation, acting as a transducer molecule for developmental processes, including segmentation and neuroblast specification | **1p36.33** | **Maternal** |
| ENSG00000162576 | *MXRA8* | Matrix Remodeling Associated 8 | Ancylostimiasis, Chikungunya.  It can modulate activity of various signaling pathways, probably via binding to integrin ITGAV:ITGB3 | **1p36.33** | **Maternal** |
| ENSG00000160075 | *SSU72* | SSU72 homolog,  RNA polymerase II CTD phosphatase | Chromatin Regulation / Acetylation | **1p36.33** | **Maternal** |
| ENSG00000178821 | *TMEM52* | Transmembrane protein 52 | Chromosome 1p36 Deletion Syndrome,  Mehmo Syndrome | **1p36.33** | **Paternal** |
| ENSG00000157916 | *RER1* | Retention in endoplasmic reticulum sorting receptor 1 | Peroxisome biogenesis disorder 1A,  Charcot-Marie-Tooth disease,  Deafness. It is involved in the retention of endoplasmic reticulum (ER) membrane proteins in the ER and retrieval of ER membrane proteins from the early Golgi compartment to facilitate gamma-secretase complex assembly | **1p36.33** | **Paternal** |
| ENSG00000157911 | *PEX10* | Peroxisomal biogenesis factor 10 | Peroxisomal biogenesis disorders 6A, 6B,  Neonatal adrenoleukodystrophy, Zellweger syndrome, Severe mental retardation. It  involved in import of peroxisomal matrix proteins | 1p36.32 | **Maternal** |
| ENSG00000157881 | *PANK4* | Pantothenate kinase 4 (inactive) | Early-onset posterior polar cataract,  Neurodegeneration with brain iron accumulation | 1p36.32 | **Maternal** |
| ENSG00000157870 | *PRXL2B*  *(C1orf93)* | Peroxiredoxin Like 2B | Among its related pathways are Arachidonic acid metabolism | 1p36.32 | **Maternal** |
| ENSG00000142611 | *PRDM16* | Histone-Lysine N-Methyltransferase PRDM16 | The reciprocal translocation t(1;3)(p36;q21) occurs in a subset of myelodysplastic syndrome (MDS) and acute myeloid leukemia (AML).  Left Ventricular Noncompaction. Among its related pathways are Respiratory electron transport, ATP synthesis by chemiosmotic coupling, and heat production by uncoupling proteins, PKMTs methylate histone lysines. | 1p36.32 | **Paternal** |
| ENSG00000162591 | *EGFL3* | Multiple Epidermal Growth Factor-Like Domains Protein 6 | It include calcium ion binding*.* | 1p36.32 | **Paternal** |
| ENSG00000116213 | *WDR8 (WRAP73)* | WD Repeat-Containing Protein Antisense To TP73 Gene | Members of this family are involved in a variety of cellular processes, including cell cycle progression, signal transduction, apoptosis, and gene regulation. | 1p36.32 | **Maternal** |
| ENSG00000078900 | *TP73* | Tumor protein p73, P53-Like Transcription Factor | Member of the p53 family of transcription factors involved in cellular responses to stress and development. Neuroblastoma, Small cell cancer of the lung, Oligodendroglioma. | 1p36.32 | **Maternal** |


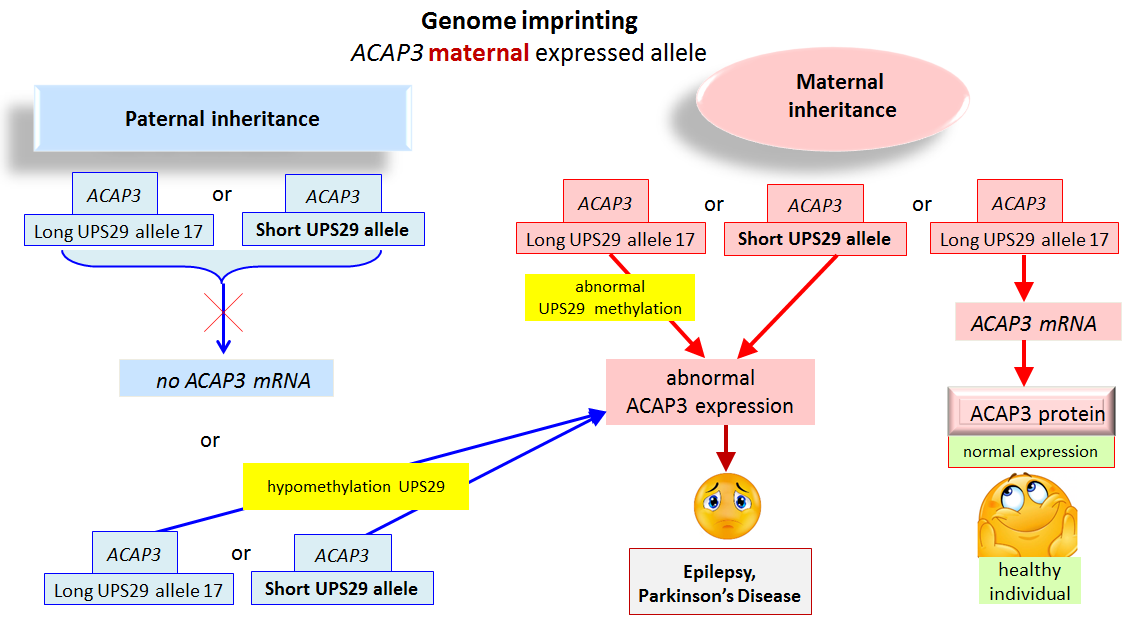


Figure S10. Hypothetical scheme explaining the cases of absence and presence of epilepsy (or Parkinson's disease) in individuals with short or long UPS29 alleles, taking into account the predicted imprinted *ACAP3* gene.

Table S7. PCR products size of the UPS29 alleles detected in the residents of St. Petersburg (Russian Federation), ethnic origin - Caucasian [Suchkova et al., 2007].

| Allele | Number of repeats | PCR fragments size of the UPS29  (promers fwRP and revPR), bp | |
| --- | --- | --- | --- |
|  |  | according to  100 bp DNA ladder  (6% PAAG) | according to sequencing results |
| 24 | 24 * | 1200 | - |
| **17 a** | 17 | 900 | 895 |
| **17 b** | 17 | 850 | 865 |
| 14 | 14 * | 750 | - |
| 10 | 10 * | 550 | - |
| **9 a** | 9 | 520 | 532 |
| **9 b** | 9 | 510 | 522 |
| **8** | 8 | 500 | 490 |
| **6 a** | 6 | 450 | 399 |
| **6 b** | 6 | 400 | 338 |
| * - non-sequenced alleles for which the estimated number of repeats is indicated (it was evaluated according to the external molecular weight standard (100 bp and 1000 bp ladders) minus 168 nt (the length of sequences flanking UPS29 including primers) and the resulting difference divided by the average repeat size (45 nt). | | | |

**References.**

Boan F., Gonzalez A. I., Rodriguez J. M., Gomez-Marquez J. Molecular characterization of a new human minisatellite that is able to form single-stranded loops in vitro and recognized by nuclear proteins. FEBS Letters. 1997. 418(3): 251-257. doi: 10.1016/s0014-5793(97)01389-6.

Weinreb A., Katzenberg D. R., Gilmore G.L., Birshtein B.K. Site of unequal sister chromatid exchange contains a potential Z-DNA-forming tract. Proc Natl Acad Sci USA. 1988. 85(20): 529-533. doi:10.1073/pnas.85.2.529.

Mar-Wiz (Detection of putative matrix attachment regions in eukaryotic DNA sequences), https://www.futuresoft.org/MAR-Wiz/, accessed 2003.

Suchkova I.O., Shubina D.M., Sasina L.K., Slominskaya N.A., Vasilyev V.B., Alenina N.V., Bader M., Patkin E.L. Molecular-genetic characteristics of the non-hypervariable GC-rich human minisatellite UPS29 of *CENTB5* gene. *Ecologicheskaya Genetika.* 2007. 5(3): *35-45.* (Article in Russian) ISSN 1811–0932.

Gururajan R.,  Lahti J.M.,  Grenet J., Easton  J.,  Gruber I.,  Ambros P.F.,  Kidd V.J. Duplication of a Genomic Region Containing the Cdc2L1-2 and MMP21-22 Genes on Human Chromosome 1p36.3 and Their Linkage to D1Z2. Genome Res. 1998. 8(9):929-939. doi: 10.1101/gr.8.9.929.

Shubina D.M., Suchkova I.O., Slominskaya N.A., Alenina N., Bader M., Patkin E.L.  CENTB5 gene expression in humans and mice. Molecular Biology. 2009.Vol.43(3):374-380. doi: 10.1134/S0026893309030042

Luedi P.P., Dietrich F.S., Weidman J.R., Bosko J.M., Jirtle R.L., Hartemink A.J. Computational and Experimental Identification of Novel Human Imprinted Genes. Genome Res. 2007. 17(12):1723-1730. doi: 10.1101/gr.6584707.

Luedi P.P., Dietrich F.S., Weidman J.R., Bosko J.M., Jirtle R.L., Hartemink A.J. Computational and Experimental Identification of Novel Human Imprinted Genes. Genome Res. 2007. 17(12):1723-1730. doi: 10.1101/gr.6584707.

## Supplemental Research Data, https://genome.cshlp.org/content/early/2007/11/30/gr.6584707/suppl/DC1
